# Unveiling shared genetic regulators of plant architectural and biomass yield traits in the Sorghum Association Panel

**DOI:** 10.1093/jxb/eraf012

**Published:** 2025-01-11

**Authors:** Anuradha Singh, Linsey Newton, James C Schnable, Addie M Thompson

**Affiliations:** Department of Plant, Soil and Microbial Sciences, Michigan State University, East Lansing, MI 48824, USA; Plant Resilience Institute, Michigan State University, East Lansing, MI 48824, USA; Department of Plant, Soil and Microbial Sciences, Michigan State University, East Lansing, MI 48824, USA; Center for Plant Science Innovation and Department of Agronomy and Horticulture, University of Nebraska-Lincoln, Lincoln, NE 68588, USA; Department of Plant, Soil and Microbial Sciences, Michigan State University, East Lansing, MI 48824, USA; Plant Resilience Institute, Michigan State University, East Lansing, MI 48824, USA; CIMMYT, Mexico

**Keywords:** Biomass yield, genome-wide association studies, plant architectural traits, pleiotropic loci, sorghum association panel

## Abstract

Sorghum is emerging as an ideal genetic model for designing high-biomass bioenergy crops. Biomass yield, a complex trait influenced by various plant architectural characteristics, is typically regulated by numerous genes. This study aimed to dissect the genetic regulators underlying 14 plant architectural traits and 10 biomass yield traits in the Sorghum Association Panel across two growing seasons. We identified 321 associated loci through genome-wide association studies (GWAS), involving 234 264 single nucleotide polymorphisms (SNPs). These loci include genes with known associations to biomass traits, such as *maturity*, *dwarfing* (*Dw*), and *leafbladeless1*, as well as several uncharacterized loci not previously linked to these traits. We also identified 22 pleiotropic loci associated with variation in multiple phenotypes. Three of these loci, located on chromosomes 3 (S03_15463061), 6 (S06_42790178; *Dw2*), and 9 (S09_57005346; *Dw1*), exerted significant and consistent effects on multiple traits across both growing seasons. Additionally, we identified three genomic hotspots on chromosomes 6, 7, and 9, each containing multiple SNPs associated with variation in plant architecture and biomass yield traits. Chromosome-wise correlation analyses revealed multiple blocks of positively associated SNPs located near or within the same genomic regions. Finally, genome-wide correlation-based network analysis showed that loci associated with flowering, plant height, leaf traits, plant density, and tiller number per plant were highly interconnected with other genetic loci influencing plant architectural and biomass yield traits. The pyramiding of favorable alleles related to these traits holds promise for enhancing the future development of bioenergy sorghum crops.

## Teaser

This study identifies shared genetic regulators and interconnected loci influencing plant architectural and biomass yield traits, enabling the pyramiding of favorable alleles for advancing bioenergy sorghum breeding efforts.

## Introduction

The inescapable depletion of fossil fuels creates an urgent need to replace them with cellulosic biofuels, which have the potential to mitigate several undesirable aspects of fossil fuel utilization, such as greenhouse gas emissions and reliance on foreign suppliers (Neupane *et al.*, 2022). Optimized bioenergy crops represent one of the most sustainable and renewable resources for plant-based biomass production (Saleem, 2022). The cultivation of bioenergy crops has been further accelerated by ethical considerations and environmental concerns (Rosegrant and Msangi, 2014; Ain *et al.*, 2022). This transition is particularly crucial given predictions that global energy demand will rise due to population growth between now and 2050 (Reid *et al.*, 2020).

Sorghum [*Sorghum bicolor* (L.) Moench], a member of the Andropogoneae tribe, constitutes the world’s fifth most important cereal crop. It is grown for both grain and forage, serving as a vital food source in many food-insecure regions around the world (Silva *et al.*, 2022). Sorghum holds great promise for lignocellulosic biomass production (Damay *et al.*, 2018; Ain *et al.*, 2022; Baloch *et al.*, 2023). It requires less water and nitrogen than maize and adapts well to a variety of soil types and environmental conditions (Lemaire *et al.*, 1996; Hirel *et al.*, 2007; Gelli *et al.*, 2014; Salehin *et al.*, 2020). Moreover, lignocellulosic ethanol produced from sorghum is projected to yield five times more net energy per unit land area than ethanol derived solely from grain starch and sugar, while emitting only a quarter of the greenhouse gases per unit energy (Mullet *et al.*, 2014). Thus, improving high-biomass sorghum could significantly boost biofuel efficiency and renewable chemical production (Baloch *et al.*, 2023).

The dry mass fraction of plant parts—such as stems, leaves, tillers, and panicles, commonly referred to as biomass—is crucial for producing lignocellulosic biofuel (Habyarimana *et al.*, 2020, 2022). Stems and leaves alone constitute 80–85% of the total harvested biomass from sorghum plants (Mullet *et al.*, 2014). Thicker stems, in particular are preferred for achieving higher biomass yields (Kong *et al.*, 2020). Various plant architectural traits of these individual parts—such as height, stem diameter and volume, leaf morphology, days to flowering, panicle length and number, and tiller propensity—are pivotal in determining the three-dimensional structure of the plant (Anami *et al.*, 2015). For instance, leaf architecture, including size, shape, weight, number, width, and length significantly influences the physiological function of plants, affecting their efficiency in capturing and utilizing sunlight, water, nitrogen, and other resources (Anami *et al.*, 2015). Flowering is critical for determining overall biomass productivity and is heavily influenced by genotype, planting location, day length, and environmental conditions. In sorghum, a delayed flowering period or extended duration of vegetative growth is a key trait associated with increased stem and leaf biomass (Rooney *et al.*, 2007). The physiological control of vegetative branching or tillering is essential in the deterministic breeding of optimized genotypes for sustainable cellulosic biomass production in both optimal and marginal conditions (Anami *et al.*, 2015). Kong *et al.* (2014) reported that both the number of tillers with mature panicles and the number of immature secondary branches consistently showed positive correlations with total dry biomass production.

Biomass yield is a composite trait influenced by multiple plant architectural traits, which are often controlled by numerous quantitative trait loci (QTLs) with larger effects (Lasky *et al.*, 2015; Miao *et al.*, 2019; Souza *et al.*, 2021). Additionally, hundreds, or even thousands, of genes from various genomic regions each contribute a small portion to the variation in biomass production among different sorghum genotypes, depending on the environmental conditions. To date, four major dwarf (*Dw*) loci (*Dw1*–*Dw4*) have been described in sorghum that regulate plant height across various environments (Quinby and Karper, 1954; Hilley *et al.*, 2016; Chen *et al.*, 2019). These dwarf loci, *Dw1*, *Dw2*, *Dw3*, and *Dw4*, have been mapped to chromosomes 9, 6, 7, and 4, respectively. The *Dw1* locus encodes a protein possibly involved in brassinosteroid signaling (Yamaguchi *et al.*, 2016), while *Dw2* encodes a protein kinase (Hilley *et al.*, 2017), *Dw3* is associated with an auxin efflux transporter (Multani *et al.*, 2003), while the causal gene for the *Dw4* locus has yet to be identified (Li *et al.*, 2015). Recently, another mutant, *dw5*, has been isolated, characterized by a single nuclear gene mutation and inherited in a recessive manner (Chen *et al.*, 2019).

Six Maturity (*Ma*) loci (*Ma1*–*Ma6*) have been identified that regulate flowering time in sorghum (Quinby and Karper, 1945; Murphy *et al.*, 2011; Casto *et al.*, 2019; Grant *et al.*, 2023). *Ma1*, located on chromosome 6, encodes a pseudo-response regulator (Murphy *et al.*, 2011). *Ma2*, found on chromosome 2, encodes a SET and MYND domain-containing lysine methyl transferase (Casto *et al.*, 2019). *Ma3* and *Ma5*, both on chromosome 1, are associated with phytochrome B (Childs *et al.*, 1997) and phytochrome C (Rooney and Aydin, 1999), respectively. *Ma4* was discovered in crosses of Milo (*Ma4*) and Hegari (*ma4*), although the underlying gene remains unidentified (Quinby, 1966). *Ma6*, located on chromosome 6, encodes Grain number, plant height, and heading date7, acting as a repressor of flowering in long days (Murphy *et al.*, 2014).

Several growth regulators, such as phytohormones, play a crucial role in plant growth and development, significantly contributing to biomass production (Sosnowski *et al.*, 2023). For instance, auxins and brassinosteroids are known to regulate plant height (Hirano *et al.*, 2017; Mu *et al.*, 2022), while gibberellin and cytokinin influence stem and leaf biomass, respectively (Skalák *et al.*, 2019; Y. Wang *et al*., 2020). The expression of primary and secondary metabolic genes, along with their associated metabolic products such as lignocellulose, sugars, starch, and various secondary metabolites, crucially impacts the bioenergy potential of sorghum biomass (Chen *et al.*, 2020; Xue *et al.*, 2023). Furthermore, the expression of these genes is frequently regulated by multiple transcription factors, and identifying these factors could provide a potential target for future molecular breeding to modulate biomass accumulation in sorghum (Scully *et al.*, 2016; Wei *et al.*, 2023).

Understanding the genetic basis of complex quantitative and polygenic traits that contribute to variation in biomass productivity is crucial for advancing the breeding of biomass sorghum cultivars. Genome-wide association studies (GWAS), which rely on genetic linkage disequilibrium, offer an opportunity to identify genes that affect the natural variation of quantitative traits by associating markers with the phenotypes of interest (Somegowda *et al.*, 2021). Several sorghum diversity panels, such as the mini-core collection (Upadhyaya *et al.*, 2009), Sorghum Association Panel (SAP) (Casa *et al.*, 2008), and Sorghum Bioenergy Panel (BAP) (Brenton *et al.*, 2016), have been extensively used for genetic dissection of multiple complex traits. For instance, the SAP has been used to study seed size traits (Zhang *et al.*, 2015), grain yield component traits (Boyles *et al.*, 2016), photosynthesis (Ortiz *et al.*, 2017), plant height (Miao *et al.*, 2020), and root system architecture (Zheng *et al.*, 2020). The BAP has been used to examine plant height (Brown *et al.*, 2008), stem sugar content and height (Murray *et al.*, 2008, 2009), flowering time (Mace *et al.*, 2013), various domestication traits (Morris *et al.*, 2013), structural and non-structural carbohydrate (Brenton *et al.*, 2016; Kumar *et al.*, 2024), and biomass accumulation under cold stress (Agnew *et al.*, 2024). Other powerful resources such as the nested association mapping (NAM) population (Bouchet *et al.*, 2017; Boatwright *et al.*, 2021), multiparent advanced generation intercross (MAGIC) population (Ongom and Ejeta, 2018; Kumar *et al.*, 2023), Ethiopian sorghum landrace collection (Girma *et al.*, 2019), and Nigerian diversity population (Maina *et al.*, 2018) have also been utilized to identify candidate genes underlying important agronomic traits. However, detailed studies investigating the genomic regions and loci associated with multiple plant architectural and biomass yield traits in any of these populations, including the SAP, are currently lacking.

The SAP was initially sequenced using simple sequence repeat markers (Casa *et al.*, 2008), followed by restriction site-based genotyping-by-sequencing (GBS) for low-coverage single-nucleotide polymorphism (SNP) data (Morris *et al.*, 2013). Subsequently, Miao *et al.* (2020) improved the SNP data quality using a modified tunable-GBS (tGBS) protocol. More recently, Boatwright *et al.* (2022) generated whole-genome sequencing data, providing high-density genomic variants such as SNPs, indels, and copy number variants. As our aim was to identify genomic regions and loci associated with multiple plant architectural and biomass yield traits in the SAP population, we performed GWAS analyses using high-confidence SNPs obtained from Miao *et al.* (2020). Fourteen plant architectural and 10 biomass yield traits were phenotyped across two seasonal growing conditions. Our analyses revealed shared genomic regions and pleiotropic loci that explained significant variability in multiple plant architectural and biomass yield traits. These findings provide valuable targets for marker-assisted selection strategies aimed at developing improved biomass sorghum cultivars.

## Materials and methods

### Plant materials, site descriptions, and growth conditions

This study utilized accessions from the SAP, which includes individuals from all five major sorghum botanical clades (*bicolor*, *caudatum*, *durra*, *guinea*, and *kafir*). These accessions were originally collected from Africa, Asia, and the Americas (Casa *et al.*, 2008) (Supplementary Table S1). The SAP accessions were grown in a randomized block design with two replications per accession across two growing seasons, spanning from June to October in 2020 (391 accessions; planted on 1 June) and 2021 (396 accessions; planted on 6 June). Each experimental plot consisted of two parallel rows, each 3 m long, separated by 76.2 cm, with 91 cm alleyways between sequential plots. Forty-one seeds per row were sown with a 7.6 cm separation, to maintain a planting density of 70 000 plants per acre. The field design for each year included multiple replicates (nearly 10% scattered checks) of the reference accession BTx623 (PI 564163) within each block (Paterson *et al.*, 2009).

Field trials were conducted under natural rainfed conditions at the Michigan State University research field (42° 42′ 53.5″ N, 84° 27′ 46.5″ W; elevation ~265 m above sea level). The soil at the research site is classified as loamy, with a high available water capacity and moderately slow permeability. After planting the seeds, weather data were monitored throughout the growing season using a weather station located approximately 16 km from the field (https://www.wunderground.com/history/monthly/us/mi/lansing/KLAN/date/2022-9). This station provided data that closely aligned with our onsite weather station, ensuring the accuracy and reliability of the collected data. The average monthly precipitation and temperature variations recorded from June to September is shown in Supplementary Fig. S1.

### Plant architectural trait phenotyping

**Fig. 1.**
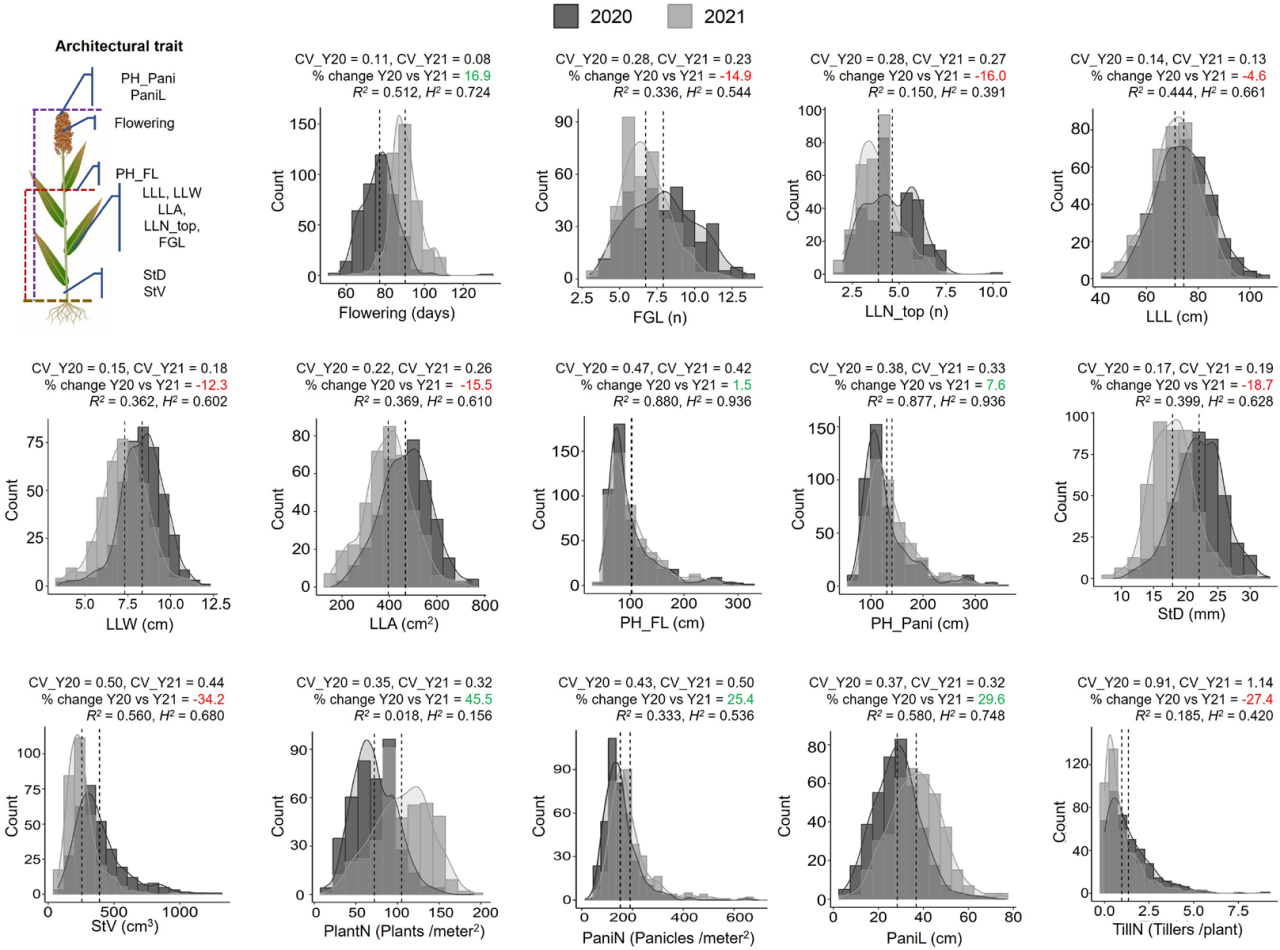
Natural variations in architectural traits. Phenotypic distribution of plant architectural traits in the Sorghum Association Panel across two growing seasons (2020 and 2021). The mean value of each trait for each growing season is depicted as dotted lines on the respective plots. The coefficient of variation for each trait in each year is denoted as CV_Y20 and CV_Y21. The percentage change in data acquisition between two growing seasons (Y20 versus Y21) is indicated, with gains shown in green and losses in red. The repeatability of data, measured through the regression coefficient (*R*^2^, *P*≤0.05) and broad-sense heritability (*H*^2^), are presented on each distribution plot. The plant architectural traits are abbreviated as follows: FGL, final green leaves; LLA, largest leaf area; LLL, largest leaf length; LLN_top, largest leaf number from top; LLW, largest leaf width; PaniL, panicle length; PaniN, panicle number per square meter; PF_FL, plant height up to flag leaf; PH_Pani, plant height up to panicle; PlantN, plant number per square meter; StD, stem diameter; StV, stem volume; TillN, tiller number per plant.

In both growing seasons (2020 and 2021), data on 14 plant architectural traits were collected. These traits included measurements related to days to flowering as well as various traits related to leaves, plant height, stem, panicles, and tillers (Fig. 1). Since these traits were manually phenotyped in the large population, data were collected from two plants per plot per growing season.

Flowering for each plot was defined as the number of days between planting and the first occurrence of anthesis in at least half of the primary panicle’s anthers on at least 50% of the plants in that plot. When the plants reached the soft-dough stage, leaf-related traits, such as the number of final green leaves (FGL) on the main stem (those with 50% or more green surface area) and the largest leaf number from the top (LLN_top), were visually marked and counted. Subsequently, the largest leaf length (LLL; cm) and largest leaf width (LLW; cm) were measured using a ruler with a precision of 0.1 cm. LLL was measured from the apex to the base of the leaf, while LLW was measured at the widest part of the leaf. Once LLL and LLW were determined, the largest leaf area (LLA; cm^2^) was calculated using the formula LLA=LLL×LLW×0.75, as described by Stickler *et al.* (1961).

At physiological maturity, defined as when 90% of the grain had attained a black layer at the base of the grain (Eastin *et al.*, 1973), plant height and stem-related traits were measured. Plant height was recorded from the base of the stalk to the apex of the panicle (PH_Pani; cm). Panicle length (PaniL; cm) was measured from the node where the flag leaf joins the stem to the apex of the panicle. Plant height up to flag leaf (PH_FL; cm) was calculated as the difference between PH_Pani and PaniL. Stem diameter (StD; mm) was measured with a digital caliper at the thickest point of the stem, typically 4–6 cm above the soil surface. Stem volume (StV; cm^3^) was calculated assuming the stem is a cylinder (*V*=π*r*^2^*h*) where *r* is StD/2) and *h* is PH_FL.

Plant density metrics were also collected as plant numbers per square meter (PlantN; plants m^−2^) by counting all the main stems using a yardstick (91.5 cm) placed in the center of each plot and dividing by the seed spacing (7.62 cm). Panicle numbers per square meter (PaniN; panicles m^−2^) were measured by counting all panicles with a yardstick placed in the center of each plot and dividing by the seed spacing. Tiller numbers per plant (TillN; *n*) were then calculated using the formula TillN=(PaniN−PlantN)/PlantN.

### Plant biomass yield trait phenotyping

**Fig. 2.**
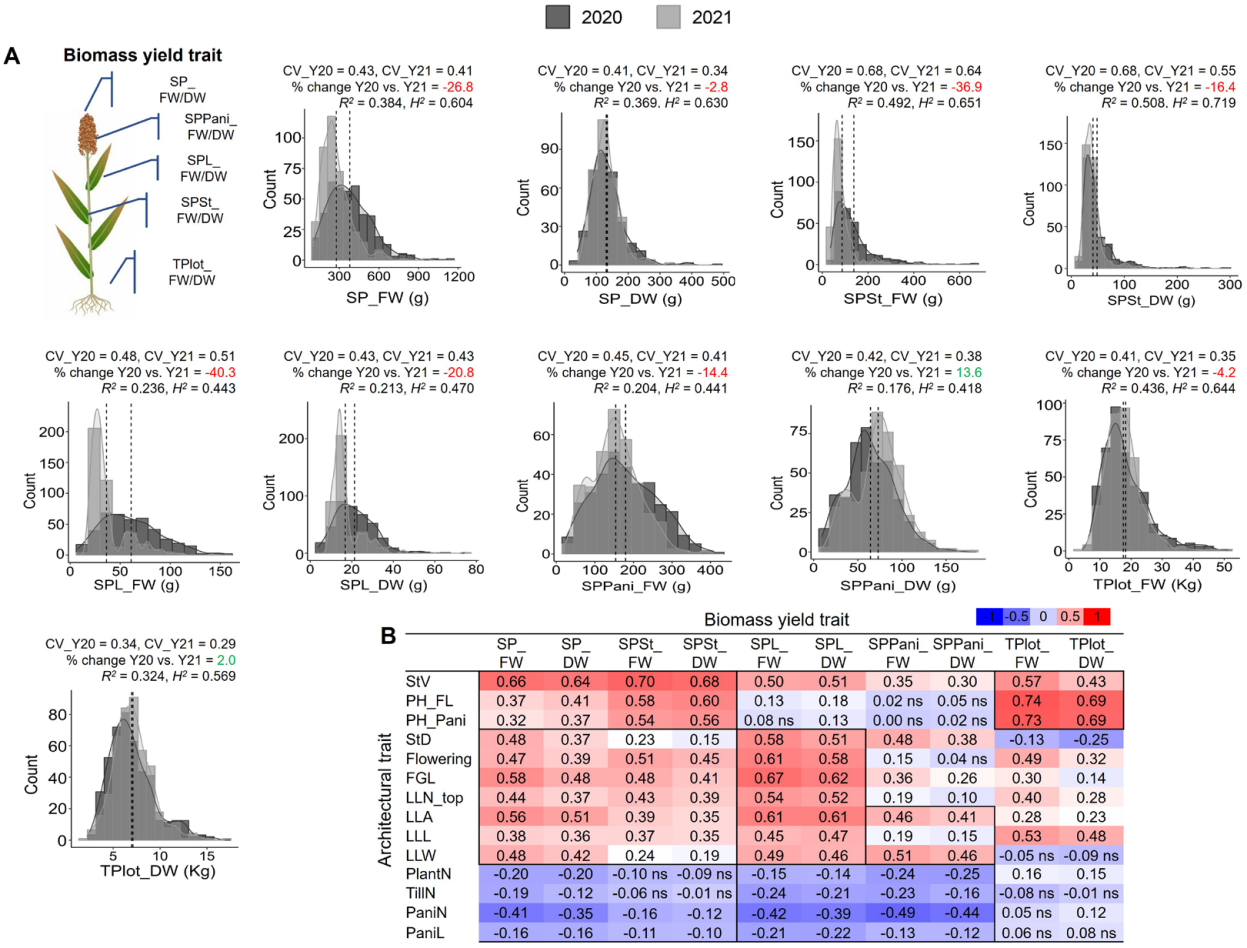
Natural variations in biomass yield traits. (A) Phenotypic distribution of plant biomass yield traits in the Sorghum Association Panel across two growing seasons (2020 and 2021). The mean value of each trait for each growing season is depicted as dotted lines on the respective plots. The coefficient of variation for each trait in each year is denoted as CV_Y20 and CV_Y21. The percentage change in data acquisition between the two growing seasons (Y20 versus Y21) is indicated, with gains shown in green and losses in red. The repeatability of data, measured through the regression coefficient (*R*^2^, *P*≤0.05) and broad-sense heritability (*H*^2^), is presented on each distribution plot. B. Pearson correlation analysis between plant architectural and biomass yield traits in the year 2020. Positive correlations are indicated in red, while negative correlations are shown in blue. All correlations, whether positive or negative, were considered significant at *P*≤0.05, with non-significant correlations denoted as ‘ns’. The biomass yield traits are abbreviated as follows: SP_DW, single plant dry weight; SP_FW, single plant fresh weight; SPL_DW, single plant leaves dry weight; SPL_FW, single plant leaves fresh weight; SPPani_DW, single plant panicle dry weight; SPPani_FW, single plant panicle fresh weight; SPSt_DW, single plant stem dry weight; SPSt_FW, single plant stem fresh weight; TPlot_FW, total plot fresh weight; TPlot_DW, total plot dry weight.

We assessed 10 biomass yield traits, eight of which were hand-harvested and two were machine-harvested. The hand-harvested biomass traits included the fresh weight (FW; g) and dry weight (DW; g) of entire single plants (SP_FW and SP_DW) as well as individual plant parts: stem (SPSt_FW and SPSt_DW), leaf (SPL_FW and SPL_DW), and panicle (SPPani_FW and SPPani_DW) (Fig. 2A). To collect these measurements, a single plant was selected from each plot, cut at the base of the stem, and its fresh weight was recorded on a digital scale (accuracy ±0.1 g). The plant was then divided into its components—stem, leaves, and panicle(s), and the fresh weights of each component were recorded. These samples were subsequently oven-dried at 60 °C for 5 d until a consistent weight was achieved. The dry weights of the stem (SPSt_DW), leaf (SPL_DW), and panicle (SPPani_DW) were recorded. The single plant dry weight (SP_DW) was then calculated as the sum SPSt_DW+SPL_DW+SPPani_DW.

For machine harvesting, a biomass harvester was used to cut the entire plot of each accession. A portion of the chopped plot was immediately weighed to obtain the sub-sample weight (SubS_FW; g). The remaining portion of the plot was weighted to determine the bucket weight. The SubS_FW and bucket weight were then combined to obtain the total plot fresh weight (TPlot_FW; kg). The sub-sample was subsequently oven-dried at 60 °C for at least 5 d, after which the dry weight of the sub-samples (SubS_DW; g) was recorded. Using the moisture content calculated from SubS_FW and SubS_DW, the total plot dry weight (TPlot_DW) was determined and expressed in kg. Since measuring detailed biomass yield traits in a large population required extensive effort, data were collected from one plant per plot per growing season.

### Spatial correction of phenotypic data

To visualize the potential confounding effect of field heterogeneity on phenotypic values, spatial analysis was conducted using a two-dimensional penalized spline method from the ‘SpATS’ library in R (Rodríguez-Álvarez *et al.*, 2018). Specifically, phenotypic data for ‘days to 50% flowering’ and ‘plant height up to flag leaf’ from the 2020 growing season were subjected to spatial analysis. As our field trial included nearly 400 accessions and multiple replicates of reference accessions grown in rows and columns, we considered rows and columns as splines and replicates as block effects. Fortunately, the phenotypic values of both traits across the accessions remained essentially unchanged even after spatial adjustment (Supplementary Fig. S2). As a result, spatial analysis for the remaining plant architectural and biomass yield traits was not conducted, and we relied on the real phenotypic values for each trait.

### Phenotypic data distribution, regression, heritability, and correlation analyses

Most statistical analyses of phenotypic data were carried out using the R statistical software (R Core Team, 2024). The phenotypic mean of each trait was calculated based on two replicates per growing season and visualized through skewed histograms using the ‘ggplot2’ (Wickham, 2016) and ‘moments’ (Komsta and Novomestky, 2015) R packages. For each growing season dataset, the Shapiro–Wilk test was performed to assess normality of the distribution. If the data did not follow a normal distribution, positive (right) skewness and negative (left) skewness were evaluated by comparing the mean and median values. Principal component analysis (PCA) of the phenotypic data from two growing seasons was performed using the ‘ggfortify’ (Horikoshi and Tang, 2018) R package. The coefficient of variation (CV) was measured as the ratio of standard deviation (σ) to mean (μ), calculated for each growing season dataset (CV=σ/μ). The repeatability of the data between growing seasons was evaluated using the regression coefficient (*R*^2^). Broad-sense heritability (*H*^2^) for all captured phenotypic data was calculated based on the impact of accession and growing season using the ‘variability’ (Popat *et al.*, 2020) R package. Each year (2020 and 2021) was treated as a distinct growing season. To analyze the effects of accession and two growing seasons on phenotypic traits, a two-way analysis of variance (ANOVA) was performed using the ‘aov()’ function in R. To determine the relationship between plant architectural and biomass yield traits, correlation analysis was performed using the phenotypic mean of the two replicates per growing season. Pearson correlations and the subsequent *P-*values were calculated using the ‘cor.test()’ function in R and plotted using Microsoft Excel.

### Genome-wide association study analyses

A genome-wide association study (GWAS) was conducted using a previously published set of 569 305 high-confidence, cleaned, and imputed SNPs genotyped across the SAP using a modified tGBS protocol (Ott *et al.*, 2017; Miao *et al.*, 2020). SNPs were scored relative to the sorghum BTx623 reference genome v3.1.1 (McCormick *et al.*, 2018), with filtering and imputation as previously described (Miao *et al.*, 2020). Markers with minor allele frequency (MAF) of less than 3% were removed, yielding 234 264 SNPs scored across 358 sorghum accessions. For each phenotypic trait, before GWAS analysis, outlier values were identified via the interquartile range (IQR; 25–75 percentile) method, and values more than 1.5 times IQR below the 25 percentile or above the 75 percentiles were removed. The ‘rMVP’ package (Yin *et al.*, 2023) in R was then used to run GWAS using the Fixed and Random Model with a Circulating Probability Unification (FarmCPU) algorithm (Liu *et al.*, 2016; Yin *et al.*, 2021). The first three principal components were fit as covariates to control population structure, and the kinship matrix computed internally by the FarmCPU algorithm was fit as a random effect (Mural *et al.*, 2021). Each GWAS model fit was assessed by examining the quantile–quantile (Q-Q) plots, and multiple trait Manhattan plots were created using the ‘CMplot’ R package (v3.6.2) (Yin, 2024). To reduce the chance of false positives, significance levels were determined using a false discovery rate (FDR)-adjusted *P*-value threshold (Benjamini–Hochberg multiple testing procedure). The phenotypic variance explained (PVE) by each SNP was calculated as described (Kumar *et al.*, 2021):


$$\begin{array}{l} PVE=\frac{2\beta^{2}MAF\times \left(1-MAF\right)}{2\beta^{2}MAF\times \left(1-MAF\right)+{\left(SE\left(\beta \right)\right)}^{2}\times 2N\times MAF\;\left(1-MAF\right)} \end{array}$$

where *N* represents the sample size of the panel, and β, SE(β), and MAF are the effect, standard error of the effect, and minor allele frequency for the genetic variant (SNP) of interest, respectively.

### Candidate gene identification and gene ontology enrichment analysis

The linkage disequilibrium decay to background levels (*r*^*2*^<0.1) in the SAP has been reported to occur in the range of 150-600 kb (Morris *et al.*, 2013). Based on this, candidate genes within 75 kb upstream and downstream of significant markers (150 kb in total) were identified using the *Sorghum bicolor* v3.1.1 database via JBrowse tool (Skinner *et al.*, 2009) on the Phytozome portal (Goodstein *et al.*, 2012; https://phytozome-next.jgi.doe.gov/). Gene ontology (GO) enrichment analysis of the identified candidate genes was then performed using PlantRegMap (Tian *et al.*, 2020; plantregmap.gao-lab.org/). A GO term was considered significantly enriched if Fisher’s exact *P*-value was <0.05. To reduce the likelihood of false positive GO terms, the FDR adjustment using the Benjamini–Hochberg multiple testing procedure at a *q*-value threshold of 0.05 was applied to determine significant GO terms.

### Correlation, haplotype, and network analysis among the SNPs

Chromosome-wise Pearson correlation analysis was conducted on the significant SNPs identified from the phenotypic data across two growing seasons. Allelic variations of each SNP were transformed into numeric values: reference allele (1), alternative allele (2), and heterozygous allele (1.5). Correlation analysis and visualization were performed using the ‘ggcorrplot’ R package (Kassambara and Patil, 2023). Haplotype analysis of selected SNPs and the visualization of the subpopulations were performed using the median-joining network in the Population Analysis with Reticulate Tree (POPART) software (Leigh and Bryant, 2015).

To identify SNPs with the most connections, genome-wide correlation-based network analysis was performed. For this, partial correlations (*r*) and *P*-values were calculated across genotype calls for all the identified SNPs in a pairwise manner using the ‘ppcor’ (Kim, 2015) R package. Then, significant positive correlated SNP pairs (*r*=0.23, *P*≤0.05) were filtered, and the correlation network was projected on Cytoscape v3.10.1 (Shannon *et al.*, 2003). The highest connectivity among SNPs was determined using the clustering coefficient algorithm in cytoHubba (Chin *et al.*, 2014).

## Results

### Overview of plant architectural and biomass yield traits: variability, heritability, and correlation analyses

In this study, we measured 14 plant architectural and 10 biomass yield traits over two growing seasons using a diverse set of sorghum accessions collected from various regions worldwide (Supplementary Table S1). These accessions exhibited substantial natural variation in their phenotypic data (Supplementary Tables S2, S3). PCA revealed that the first two principal components explained 48.3% of the overall variation (Supplementary Fig. S3). Among the 48 traits measured (24 per growing season), seven traits followed a normal distribution, while 36 traits exhibited positive skewness and five traits showed negative skewness (Figs 1, 2A; Supplementary Table S4).

Significant variations in temperature and precipitation were observed between two growing seasons at the university research field. Specifically, temperature and precipitation increased notably in 2021, rising by almost 4.5% and 52%, respectively, resulting in a warmer and more humid year (Supplementary Fig. S1). These seasonal variations led to observable differences in plant architectural and biomass yield traits. For instance, PH_Pani, days to 50% flowering, PlantN, and PaniN were overestimated by 7.6–45.4% in 2021 compared with 2020, while all leaf- and stem-related traits were underestimated by 4.6–34.2% (Fig. 1). The variation in architectural traits affected biomass yield traits, with most being underestimated in the following year compared with the previous year (Fig. 2A).

We tested the effects of accession and growing season on the plant architectural and yield traits using a two-way ANOVA. Significant effects of accession on the phenotyped traits were observed (Supplementary Table S5). Additionally, growing season had a significant impact on most phenotypic traits, except for three specific traits (PH_FL with a 1.45% change, SP_DW with a −2.79% change, and TPlot_DW with a 2.04% change), where the percentage changes in data collections were relatively modest and not significant (Figs 1, 2A). We then used the CV to compare the phenotypic plasticity of traits across growing seasons. Among plant architectural traits, flowering exhibited the lowest phenotypic plasticity, while TillN showed the highest in both growing seasons, supporting previous findings (Kim *et al.*, 2010). Biomass yield traits exhibited modest plasticity, ranging from 0.29 (TPlot_DW) to 0.68 (SPSt_FW/DW), indicating that plant architectural traits underlying biomass yield traits remained stable across growing seasons (Figs 1, 2A).

The phenotypic data on plant architectural and biomass yield traits from the two growing seasons exhibited strong repeatability, with the highest regression coefficient (*R*^2^) observed for PH_FL (0.88) and the lowest for PlantN (0.018). The *R*^2^ values for biomass yield traits were moderate, ranging from 0.18 (SPPani_DW) to 0.51 (SPSt_DW). Broad-sense heritability (*H*^2^) for plant architectural traits ranged from 0.156 (PlantN) to 0.936 (PH_FL), while biomass yield traits showed moderate heritability, with a range of 0.418 (SPPani_DW) to 0.630 (SP_DW) (Figs 1, 2A). We also conducted Pearson correlation analysis between plant architectural and biomass traits, separately for each growing season (Fig. 2B; Supplementary Fig. S4). A similar correlation pattern was observed in both season’s phenotypic data; however, the magnitude of correlation coefficients was slightly reduced in 2021 (Fig. 2B; Supplementary Fig. S4). Most plant architectural traits, excluding PlantN, TillN, PaniN, and PaniL, exhibited significant positive correlations with biomass yield traits. For instance, plant height and stem volume showed strong positive correlations with biomass traits such as SP_FW/DW, SPSt_FW/DW, and TPlot_FW/DW. Although stem volume is derived from both plant height and stem diameter, plant height contributed more substantially to the equation due to its larger magnitude compared with the square of stem diameter. As a result, plant height appeared to be a more significant determinant of total plot biomass than stem volume. Additionally, flowering and leaf architectural traits were positively associated with leaf biomass, likely due to the extended vegetative growth of plants. Conversely, negative correlations were found between all biomass yield traits and PlantN, TillN, PaniN, and PaniL, indicating competition for resource utilization (Yang *et al.*, 2019; Burgess and Cardoso, 2023). Overall, plant architectural traits were identified as key drivers of biomass accumulation in sorghum.

### Genome-wide association studies on plant architectural and biomass yield traits in sorghum

We conducted independent GWAS on 14 architectural and 10 biomass yield traits collected over two growing seasons. The FarmCPU method was employed to detect significant SNP–trait associations due to its favorable trade-off between power and FDR for moderately complex traits, and its increased capability to identify rare causal variants (Miao *et al.*, 2019). Manhattan plots and Q-Q plots, which illustrate observed *P-*values exceeding the expected *P*-values, for all traits are shown in Supplementary Fig. S5.

A total of 321 significant SNPs were detected across the two growing seasons (Supplementary Fig. S6A; Supplementary Table S6). Approximately 38% of these SNPs were located on chromosomes 6, 4, and 9, while the remaining 62% were spread across other chromosomes (Supplementary Fig. S6B). Among these SNPs, 101 fell within the cutoff range of 1.44×10^−40^ to 9.75×10^−9^ (*P-*value), while the remaining 220 were in the cutoff range of 1.10×10^−8^ to 2.4×10^−6^ (Supplementary Fig. S6C, Supplementary Table S6). In the 2020 growing season, a total of 161 SNPs were detected for a total of 23 traits, except for PlantN, which had no significant SNPs (Supplementary Fig. S6D). In that year, leaf-related traits (LLL and LLW) exhibited the highest number of detected SNPs, while SP_FW had only one significant associated SNP. In 2021, excluding TPlot_FW/DW traits, a total of 160 SNPs were identified across 22 traits, with StV having the highest number of SNPs and Plant_N and SP_DW having the fewest (Supplementary Fig. S6D).

The phenotypic variance explained (PVE) by each SNP varied considerably. The minimum PVE (PVE_min) ranged from 2.5% (for TPlot_DW by S06_36711969) to 6.5% (for PlantN by S10_50508696), while the maximum PVE (PVE_max) ranged from 6.9% (for TPlot_DW by S06_30488539) to 38.8% (for PH_Pani, S09_57005346). Notably, SNP S09_57005346 exhibited the greatest PVE_max not only for PH_Pani, but also for PH_FL (32.05%), StV (20.95%), and SPSt_DW (12.25%) (Supplementary Fig. S6D; Supplementary Table S6).

We then extracted candidate genes centered on each significant SNP within a 150 kb range (75kb upstream and downstream of each SNP). In total, approximately 2700 candidate genes were identified (Supplementary Table S7). These genes were predominantly associated with biological pathways related to ‘single organism process’, ‘response to stimulus’, ‘multicellular organismal development’, ‘anatomical structure development’, ‘gene expression’, and ‘macromolecule biosynthetic process’ (Supplementary Fig. S7). Detailed descriptions of these genes and their connections to plant architectural and biomass yield traits are provided below.

### Genetic analysis of plant architectural and biomass yield traits revealed both known and novel regulators

#### Days to flowering

**Fig. 3.**
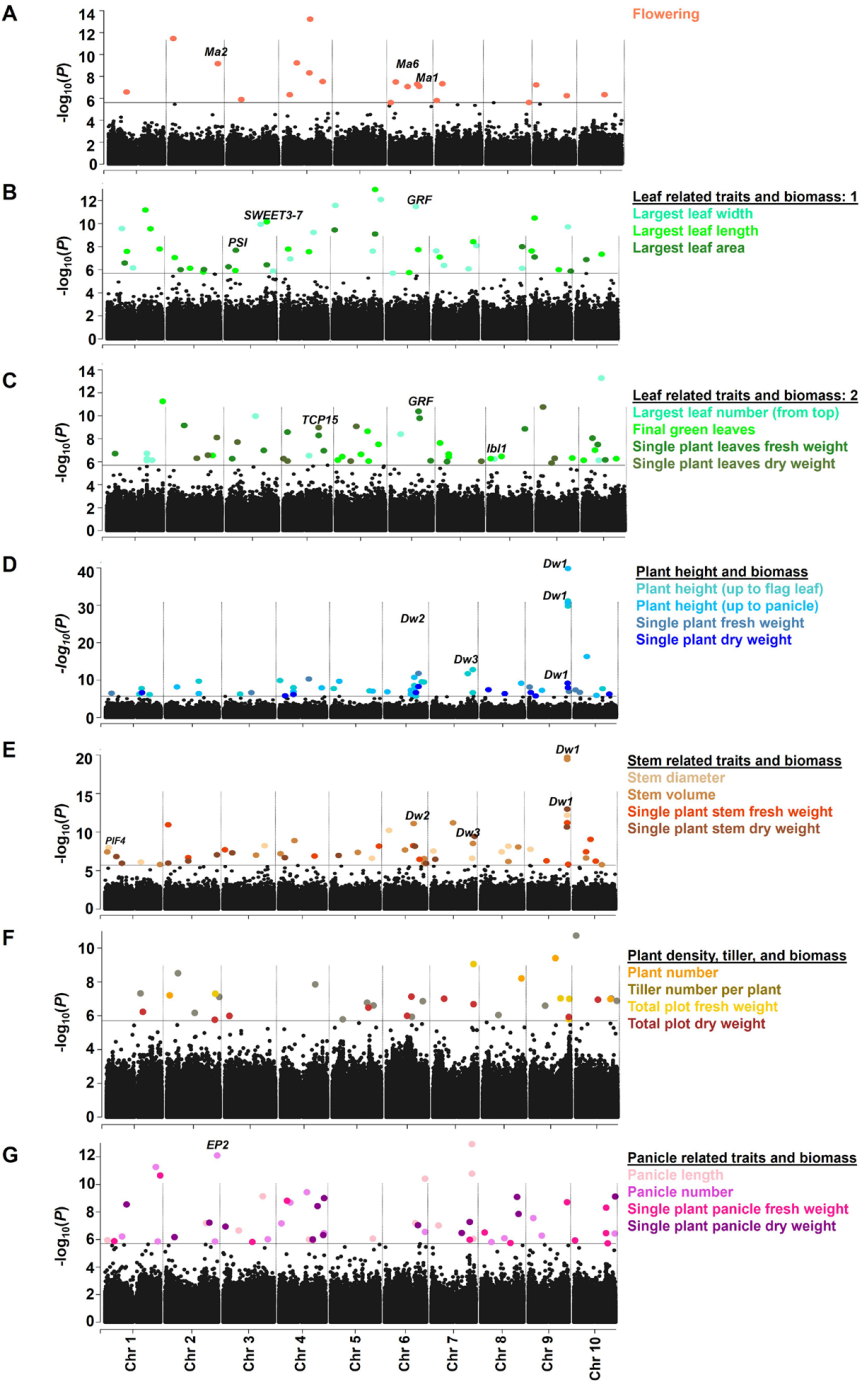
A Manhattan plot showing significant SNPs from genome-wide association studies (GWAS) for plant architectural and biomass yield traits in the Sorghum Association Panel across two growing seasons. A total of 321 SNPs were identified using the FarmCPU-based GWAS method. The plot shows −log10(*P*-values) of SNPs on the *y*-axis, with SNPs ordered by chromosomal location on the *x*-axis. A grey line indicates the significance threshold (2.4×10^−6^). For a total of 24 traits, multiple Manhattan plots (A–G) are presented, with SNPs color-coded according to the respective traits. Known genes are marked on the plot. Abbreviations: *Dw, Dwarfing*; *EP2*, *Erect Panicle2*; *GRF*, *GROWTH-REGULATING FACTOR*; *lbl1*, *leafbladeless1*; *Ma*, *Maturity*; *PSI*, *Photosystem I*; *SWEET3-7*, *Sugars Will Eventually be Exported Transporters*; *TCP*, *TEOSINTE BRANCHED1*, *CYCLOIDEA*, *and PROLIFERATING CELL FACTORS.*

Days to 50% flowering is a pivotal agronomical trait, influencing both plant size and biomass at maturity (Habyarimana *et al.*, 2020). A total of 20 SNPs associated with flowering were identified, located near 167 candidate genes (Fig. 3A; Supplementary Table S8). Six maturity loci (*Ma1–Ma6*) have been recognized as key regulators of flowering time in sorghum (Quinby, 1966; Murphy *et al.*, 2011; Casto *et al.*, 2019). Our GWAS analyses confirmed the previously reported *Ma1* (S06_40580184; T/G), *Ma2* (S02_71011504; T/C), and *Ma6* (S06_26501314; T/C) loci. The alternative alleles of these SNPs were associated with a flowering delay of approximately 2.5–3.5 d compared with reference alleles in sorghum accessions. Notably, the co-occurrence of *Ma1* and *Ma6* loci on the same chromosome further exacerbated this delay, resulting in a significant extension of flowering time by 6–13 additional days across sorghum accessions with various alternative allele combinations (Supplementary Fig. S8).

Our GWAS analyses also uncovered previously unrecognized regulators of flowering time based on SNP effects and PVE. For instance, the significant SNP S04_38064121 exhibited the most pronounced effect on flowering, with accession carrying the alternative alleles flowering approximately 4.5 d earlier. The nearest gene, located beyond the linkage disequilibrium region, was identified as a ZINC FINGER CCHC domain containing protein. Another significant SNP, S04_39191115, exhibited the highest PVE of 12.3% and was located near a gene encoding a tetratricopeptide-like helical domain (Supplementary Tables S6, S8). We also identified five potential regulatory candidates of flowering time such as zinc ion binding protein, ARF GTPase-activating domain-containing protein, glyoxylate reductase, RNA-dependent RNA polymerase, and glyoxalase, with SNPs co-localized within their genomic regions (Supplementary Table S8). Our findings suggest that these genes may be potential candidates for regulating flowering time, which require further investigation to elucidate their specific impacts and mechanisms.

#### Leaf-related traits and corresponding biomass yield traits

Leaf-related traits, including size, number, and stay-green characteristics, are crucial for plant fitness and adaptation to environmental conditions (Yin *et al.*, 2022). Our study identified 103 SNPs associated with seven leaf-related traits: FGL, LLN_top, LLL, LLW, LLA, SPL_FW, and SPL_DW (Fig. 3B, 3C; Supplementary Table S6). These SNPs were located near approximately 800 genes, 14 of which had SNPs within their genomic sequence (Supplementary Table S9). The identified genes were involved in various biological processes, including photosynthesis, metabolite biosynthesis, phytohormone biosynthesis/degradation, and transcription factor regulation. One notable candidate gene, Sobic.008G070700, an orthologue of maize *leafbladeless1* (*lbl1*, Zm00001eb264310), was associated with SNP S08_9353971, which was linked to LLN_top (largest leaf number, counted from top). The *lbl1* gene is known to influence a variety of leaf and plant phenotypes, including the specification of adaxial cell identity within leaves and leaf-like lateral organs (Nogueira *et al.*, 2007).

Photosynthesis is vital for biomass production, and leaf traits are key determinants of this process (Li *et al.*, 2021). Two SNPs, S03_4722073 and S10_18278152, associated with LLA and FGL, respectively, co-localized with photosystem I reaction center subunit XI (Sobic.003G052500) and thioredoxin (Sobic.010G132100; SNP within genomic sequence). The products of photosynthesis are subsequently converted into primary and secondary metabolites (Johnson, 2016). Our analysis identified several metabolic genes involved in carbohydrate metabolism (fructokinase-1, pyruvate kinase, oxidoreductase, epimerase, transketolase, starch branching enzyme), lipid metabolism (Sobic.001G344400, Sobic.006G034200), flavonoid biosynthesis (Sobic.001G360700, Sobic.005G033801, Sobic.005G135700, Sobic.002G337400), isoprene production (Sobic.007G196900), and both structural and non-structural polysaccharide biosynthesis (Sobic.003G052300, Sobic.001G224300, Sobic.004G075900, Sobic.006G066800). These genes are likely involved in sorghum biomass regulation by participating in primary and secondary metabolic pathways (Chen *et al.*, 2020).

We also identified several pleiotropic loci that influence multiple leaf-related and biomass yield traits (Supplementary Table S6). For instance, SNP S03_60633978, associated with LLL and LLA, was located near *SbSWEET3-7* (Sugars Will Eventually be Exported Transporters; Sobic.003G269300), a photosynthate transporter that regulates photosynthate content in different tissues (Mizuno *et al.*, 2016). This transporter contributes to the structural component of leaf biomass (Li *et al.*, 2021). Another significant locus, S03_15463061, associated with LLA and SPL_DW, was adjacent to genes encoding pectinesterase (Sobic.003G148300, Sobic.003G148400) and endoglucanase (Sobic.003G148500). These enzymes are important for cell wall modification and biomass accumulation (Tavares *et al.*, 2015; Wu *et al.*, 2018).

Transcription factors also play a critical role in leaf biomass (Zhang *et al.*, 2017; Wei *et al.*, 2023). TEOSINTE BRANCHED1, CYCLOIDEA, and PROLIFERATING CELL FACTORS (TCP) are known as growth suppressors, involved in shaping leaf and plant architecture (Nicolas and Cubas, 2016). In our study, SNP S04_58517971, which co-localized with TCP (Sobic.004G237300: SNP within genomic sequence), negatively impacted leaf biomass (Supplementary Fig. S9). In contrast, the locus S04_6235481, which co-localized with several PLANT A/T-RICH SEQUENCE- AND ZINC-BINDING PROTEINS (Sobic.004G076400, Sobic.004G076600, Sobic.004G076666), exhibited a positive impact on leaf biomass (Supplementary Fig. S10). Furthermore, SNP S05_61965692, which displayed pleiotropic effects on LLW and LLA, explained the highest PVE (13.5%). This SNP was located near *SbGRF6* (GROWTH-REGULATING FACTOR; Sobic.005G150900), although it did not significantly impact leaf biomass (Supplementary Fig. S11).

#### Plant height, stem diameter, stem volume, and corresponding biomass traits

Plant height is a crucial agronomic trait affected by several factors, including the length of each internode, the rate of internode production, and the duration of vegetative growth (Hilley *et al.*, 2016). Stem diameter also plays a significant role in maintaining the erect posture of plants and reducing lodging (Shah *et al.*, 2019). Both elongated internode and stem diameter contribute to stem volume and overall whole plant and stem biomass yield. Our study identified 114 SNPs associated with eight traits: PH_FL, PH_Pani, StD, StV, SP_FW, SP_DW, SPSt_FW, and SPSt_DW (Fig. 3D, 3E; Supplementary Table S6). Notably, 14 of these SNPs exhibited pleiotropic effects, regulating more than one trait as shown in Supplementary Table S6. These 114 SNPs were close to approximately 900 genes, which were involved in primary and secondary metabolism and hormone biosynthetic or signaling pathways genes (Supplementary Table S10).

Plant height is regulated by four dwarf loci (*Dw*), denoted *Dw1*–*Dw4*, which control internode length (Quinby and Karper, 1954; Chen *et al.*, 2019). Our GWAS analyses consistently identified the *Dw1* locus across both growing seasons, associated with SNP S09_57005346. We also identified *Dw2* and *Dw3* loci, associated with S06_42790178 and S07_59944757 SNPs, respectively. Another plant height locus, identified near the *Dw3* locus, was associated with S07_52737620 (Li *et al.*, 2015). The gene underlying this SNP was found to be *TASSELSEED2* (Sobic.007G123000), which encodes a short-chain alcohol dehydrogenase. This gene was shown to cause abortion of the gynoecium in staminate flowers of maize (DeLong *et al.*, 1993), while *tasselseed2* mutants exhibited reduced plant height with all female florets (Irish *et al.*, 1994).

Our GWAS analyses also identified several emerging regulators of plant height and stem-related traits, which were primarily based on SNP PVE. Notably, the SNP associated with the *Dw1* locus (S09_57005346) exhibited the highest range of PVE, from 12.3% to 38.9% across multiple traits (Supplementary Table S6). In addition to the *Dw1* locus, SNPs associated with StD (S09_56843188; 12.1%), StV (S07_32152737; 11.9%), SP_FW (S06_48856570; 11.5%), SPSt_FW (S02_4477084; 11.3%), and SP_DW/SPSt_DW (S09_56542041; 9.3–10.7%) showed high PVE values. The neighboring genes around these SNPs were related to macromolecule metabolism. For instance, genes related to cellulase (Sobic.006G122200 and Sobic.006G122300) and wax synthase (Sobic.006G123200 and Sobic.009G226600) are involved in maintaining carbohydrate and lipid levels in the vegetative tissue (mainly stem) of sorghum plants, as shown previously (McKinley *et al.*, 2022). Our study also identified 21 genes with significant SNPs in their genomic regions. Some of these genes were annotated as histone deacetylase, phosphate-transporting ATPase/ABC phosphate transporter, UDP-glycosyltransferase, and many more (Supplementary Table S10). These genes are believed to regulate both whole-plant and stem biomass yield in sorghum, warranting further investigation.

#### Tiller number per plant, plant numbers per square meter, and total plot biomass

A total of 33 SNPs were identified across the four traits TillN, PlantN, TPlot_FW, and TPlot_DW. Among them, 15, 4, 4, and 10 SNPs were associated with TillN, PlantN, TPlot_FW, and TPlot_DW, respectively (Fig. 3F; Supplementary Table S6). These SNPs were close to 289 genes, including six genes with significant SNPs within their genomic sequences (Supplementary Table S11). The identified genes were associated with phytohormones, transcription factors, and plant cell wall degrading enzymes (Supplementary Table S11).

Tillering, or vegetative branching, is one of the most plastic traits affecting both biomass yield and grain yield in many crop plants (Kim *et al.*, 2010). A SNP associated with TillN (S10_562834) had the greatest PVE (10.1%). This SNP was located near auxin-induced protein 5NG4 (Sobic.010G006700 and Sobic.010G006800) and invertase/pectin methylesterase (Sobic.010G006900 and Sobic.010G007600). These genes have been previously shown to regulate tiller number and influence the biochemical composition of cell wall in rice (Chen *et al.*, 2012; Nguyen *et al.*, 2017).

Tiller number is also influenced by planting density (Yang *et al.*, 2019). In our study, one SNP related to PlantN (S08_56617080, PVE 7.4%) was adjacent to the MYB transcription factor (Sobic.008G137500) (Supplementary Tables S6, S11), a gene known to regulate tiller growth in rice (F. Wang *et al.*, 2020). Another significant SNP (S07_59146937) turned out to be pleiotropic for both TPlot_FW and TPlot_DW (Supplementary Table S6). This SNP was located near genes related to phytohormone receptors, specifically for gibberellin and brassinosteroid, and carbohydrate metabolic genes, including epimerase and α-amylase. Previous studies have suggested that gibberellin biosynthesis is regulated by brassinosteroids, which together enhance biomass yield in maize (Hu *et al.*, 2017).

#### Panicle number per square meter, panicle length, and corresponding biomass yield traits

A total of 51 SNPs were identified across the four traits of PaniN, PaniL, SPPani_FW, and SPPani_DW. Specifically, 11, 16, 8, and 15 SNPs were found to be associated with PaniL, PaniN, SPPani_FW and, SPPani_DW, respectively (Fig. 3G; Supplementary Table S6). These 51 SNPs were positioned near 533 genes, 15 of which had SNPs within their genomic sequences (Supplementary Table S12). The identified genes were involved in various metabolic pathways such as carbohydrate (epimerase, transketolase, trehalose-6-phosphate synthase, xylose isomerase, lactate/malate dehydrogenase), lipid (acyl-desaturase, diacylglycerol kinase, esterase, lipid phosphatase protein, myristoyl-acyl carrier protein thioesterase), and flavonoid biosynthesis (anthocyanidin 5,3-*O*-glucosyltransferase, naringenin, 2-oxoglutarate 3-dioxygenase), hormones (auxin-responsive Aux/IAA, flavin-containing monooxygenase family protein), and transcription factors (WRKYs, MYBs, AP2, NAC, and Homeodomain) (Supplementary Table S12).

Interestingly, none of these genes showed homology to known panicle-related genes, suggesting the involvement of emerging regulators that may be influencing sorghum panicle traits. Notably, a locus related to PaniN (S02_72662765) was located near Sobic.002G374400 gene, beyond the linkage disequilibrium range (0.5 Mb downstream). This gene shares ~69% amino acid sequence identity with Erect Panicle2 (EP2) in indica rice and encodes a novel plant-specific protein localized to the endoplasmic reticulum with unknown function. The *ep2-1* and *ep2-2* mutants in rice exhibit shorter panicle length, more vascular bundles, and a thicker stem than that of wild-type plants, resulting in an erect panicle phenotype (Zhu *et al.*, 2010). Given that panicle architecture and grain development are highly conserved between rice and sorghum (Chen *et al.*, 2015), this gene could be a candidate for regulating panicle morphology in sorghum, requiring more functional studies.

### Plant architectural and biomass yield traits as regulated through shared hub genomic regions and pleiotropic loci

**Fig. 4.**
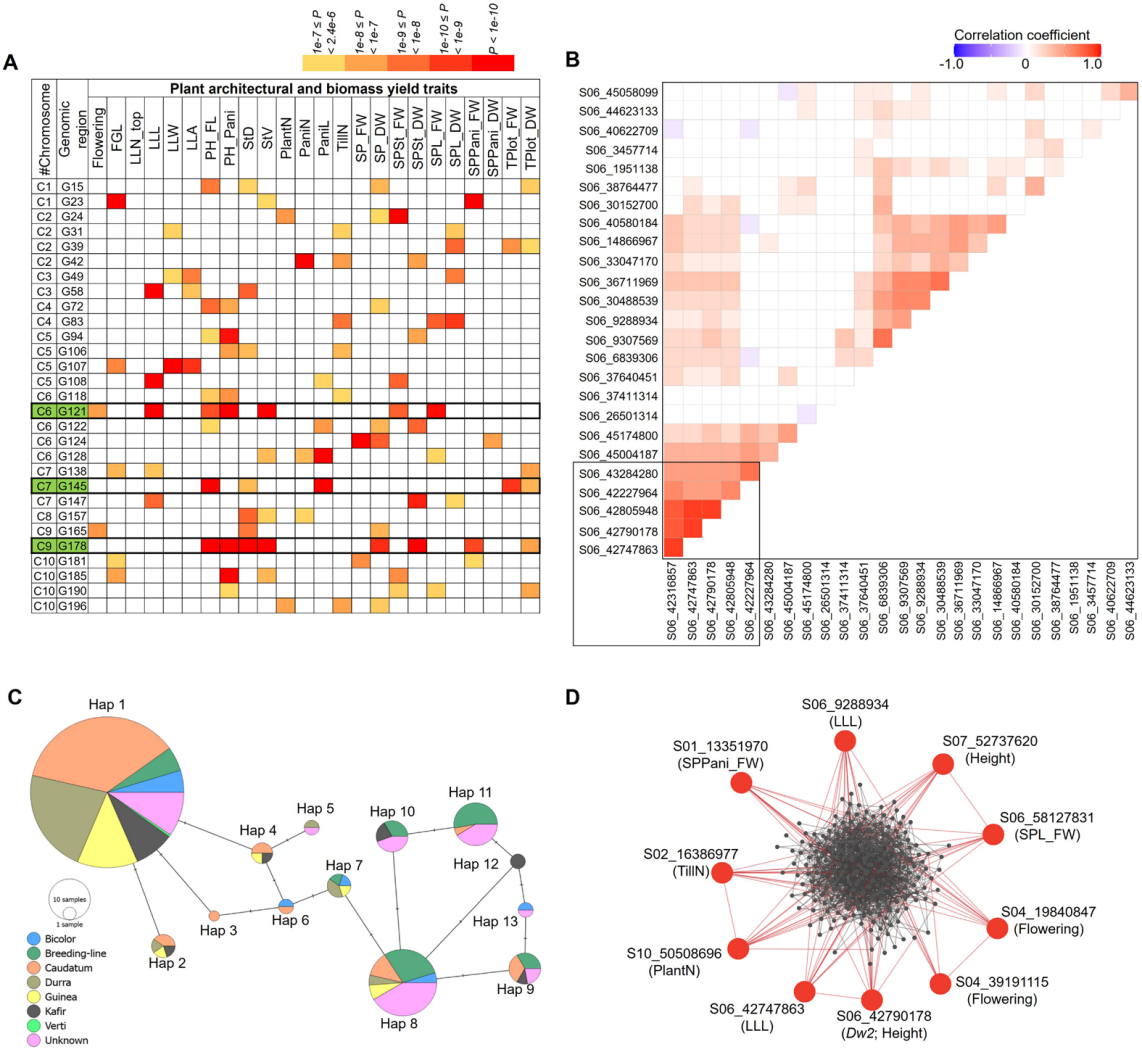
Genomic region visualization, correlation analysis, haplotype analysis, and identification of highly connected SNPs for multiple plant architectural and biomass yield traits in the Sorghum Association Panel. (A) Distribution of significant SNPs in 29 genomic regions associated with at least three traits (including plant architecture or biomass or both). These genomic regions are numerically coded, with details provided in Supplementary Table S13. The significance of each SNP is color-coded based on the *P-*value of the association, with white boxes indicating no significant association. Solid boxes with green highlighting around specific genomic regions denote hotspot areas exhibiting multiple SNPs associated with plant architecture and biomass yield traits. (B) Chromosome-wise Pearson correlation analysis among significant SNPs identified from phenotypic data across two growing seasons. The figure focuses on chromosome 6 SNPs. Positive correlations are indicated in red, while negative correlations are shown in blue. All correlations, whether positive or negative, were considered significant at *P*≤0.05, with non-significant correlations denoted as white. The black box highlights the SNP blocks with positive associations. (C) Haplotype analysis of six SNPs from the genomic region 121 (42227964–43284280) on chromosome 6. A total of 13 distinct haplotype variants were identified. These haplotype variants were further explored at the level of their distribution among different sorghum races and their impact on phenotypic values. (D) Identification of the highly connected SNPs through genome-wide correlation-based network analysis among all SNPs in a pairwise manner. The network was visualized using Cytoscape and the highest connectivity among SNPs was determined using the clustering coefficient algorithm in cytoHubba.

Multiple plant architectural traits exhibited significant correlations to biomass yield traits at the phenotypic level across both growing seasons. This led us to hypothesize that these traits might share common molecular regulators at the genetic level. To illustrate this, we mapped 321 significant SNPs onto the sorghum genome at 600 kb intervals, termed genomic regions (GR). In total, 198 GR spanning all 10 chromosomes were identified (Supplementary Fig. S12; Supplementary Table S13). Among these, 29 regions were associated with three or more traits (either plant architecture or biomass or both). Notably, three hotspot regions, GR_121 on chromosome 6 (42227964–43284280), GR_145 on chromosome 7 (59146937–59746937), and GR_178 on chromosome 9 (56113408–56713408), co-localized with almost all the plant architecture and biomass yield traits (Fig. 4A). We also identified several pleiotropic loci that regulate multiple plant architectural and biomass yield traits (Supplementary Table S6). Specifically, 13 pleiotropic loci were detected in the first growing season (2020), and eight in the second season (2021). Among these, 12 loci from 2020 and six from 2021 were predominantly co-localized with two different traits. Notably, locus S10_18255972 from the 2021 growing season regulated three traits together (SPSt, PH_Pani, and StV). Additionally, three pleiotropic loci were conserved across both growing seasons. For instance, locus S03_15463061 regulated multiple leaf-related traits and was close to cell wall degrading genes such as endoglucanase and pectinesterase. Genetic loci S06_42790178 and S09_57005346 linked to the *Dw*2 and *Dw*1 genes, respectively, regulated multiple traits related to plant height, stem volume, and related biomass yield traits (Hilley *et al.*, 2016, 2017).

### Pairwise correlation analysis of significant SNPs revealed blocks of highly correlated markers

We next performed a chromosome-wise Pearson correlation analysis among the identified significant SNPs. Within each chromosome, multiple pairs of highly correlated markers (*r*≥0.85) were identified (Supplementary Fig. S13). For instance, three SNPs associated with LLN_top (S01_57545826, S01_57552937, and S01_57563841), a pair of SNPs associated with PH_FL and PH_Pani (S04_20578520 and S04_20578555), and another pair of SNPs regulating FGL and StD (S07_3517563 and S07_3522065) showed higher correlation with each other (Supplementary Fig. S13). As these highly correlated SNPs were in the same genomic region, they can be considered as alternative haplotypes.

We also identified several SNP blocks on chromosomes 3, 6, 7, 8, 9, and 10, which, although belonging to different genomic regions, exhibited moderate to high correlation (*r*>0.50) (Fig. 4B; Supplementary Fig. S13). Since correlation analysis identified highly correlated SNP blocks, we performed haplotype analysis on six SNPs: S06_42227964 (G/T), S06_42316857 (C/T), S06_42747863 (A/T), S06_42790178 (*Dw2*; T/C), S06_42805948 (T/C), and S06_43284280 (T/C). The aim of this analysis was to visualize the impact of allelic combinations on the associated traits (Fig. 4C). A total of 13 haplotype combinations were identified (Table 1). Among them, haplotype 1 emerged as the largest (211 accessions), comprising the reference alleles (GCATTT) for all the selected SNPs and mostly represented by *caudatum*, *durra*, and *guinea* races (Fig. 4C). Haplotype 8 was the second largest (41 accessions), carrying four alternative alleles (GTTCCT) and was predominantly represented by breeding lines. Haplotype 11, the third largest (17 accessions), with alternative alleles (TTTCCC) for all the selected SNPs, was also primarily represented by breeding lines. Noticeable differences were observed in the quantified data of SPL_FW, SPSt_FW, LLL, PH_FL, StV, and flowering as transitions occurred from haplotype 1 to haplotype 11 (Table 1). More specifically, haplotype 1 exhibited lower magnitudes for the quantified traits compared with haplotype 11, which had alternative alleles for all the SNPs. This resulted in delayed flowering and increased plant stature, which collectively affect several biomass yield traits, as previously shown by Higgins *et al.* (2014).

**Table 1.** Haplotype analysis and mean trait values across haplotypes.

Haplotype number	Haplotype sequence	No. of accessions	SPL_FW (g)	SPSt_FW (g)	LLL (cm)	PH_FL (cm)	StV (cm^3^)	Flowering (days)
**Hap 1**	**GCATTT**	**211**	**47.7** ^**b**^	**97.5** ^**c**^	**70.7** ^**d**^	**89.3** ^**c,d**^	**292.6** ^**b,c**^	**83.2** ^**a,b**^
Hap 2	GCACTT	5	48.4^a,b^	158.6^a,b^	76.3^b,c^	140.1^a^	456.9^a^	87.4^a^
Hap 3	GCTTTT	1	36.9^a,b^	76.8^c,d^	87.6^a,b^	113.9^b,c^	407.7^a,b^	79.0^a,b^
Hap 4	GTATTT	4	34.2^b^	65.7^d^	70.0^c,d^	87.6^c,d^	241.1^b,c^	81.1^a,b^
Hap 5	TTATTT	2	52.1^a,b^	91.4^c,d^	75.2^b,c^	62.6^d^	285.8^b,c^	86.6^a^
Hap 6	GTTTTT	2	38.5^a,b^	98.5^c^	74.4^c,d^	78.5^c,d^	228.6^b,c^	82.9^a,b^
Hap 7	GTTCTT	5	60.9^a,b^	177.5^a^	68.3^d^	106.4^b,c^	238.7^b,c^	78.4^a,b^
**Hap 8**	**GTTCCT**	**41**	**56.5** ^**a**^	**140.4** ^**a,b**^	**77.4** ^**b,c**^	**127.6** ^**a,b**^	**401.9** ^**a,b**^	**85.1** ^**a**^
Hap 9	GCTCCT	9	44.8^a,b^	89.6^c,d^	75.3^b,c^	92.3^c,d^	317.5^b,c^	82.2^a,b^
Hap 10	TTTCCT	9	45.8^a,b^	107.9^b,c^	71.8^c,d^	114.5^b,c^	320.1^b,c^	82.4^a,b^
**Hap 11**	**TTTCCC**	**17**	**59.4** ^**a**^	**118.6** ^**b,c**^	**79.2** ^**a,b**^	**94.0** ^**c,d**^	**337.6** ^**a,b**^	**85.9** ^**a**^
Hap 12	GTTCCC	2	35.6^a,b^	95.4^c^	83.9^a,b^	136.4^a,b^	370.4^a,b^	88.8^a^
Hap 13	GCTCCC	2	32.5^a,b^	113.3^b,c^	69.3^c,d^	120.9^a,b^	277.9^b,c^	83.9^a^

This analysis was conducted using SNPs from genomic region 121 (42227964–43284280) on chromosome 6. Haplotype (Hap) 1, 8, and 11 are highlighted in bold. The table presents the mean trait values of accessions carrying different allele combinations. Different letters by each value indicate significant differences among the haplotypes, as determined by one-way ANOVA with Tukey’s HSD post-hoc test at *P*≤0.05. Trait abbreviations: LLL, largest leaf length; PH_FL, plant height up to flag leaf; SPL_FW, single plant leaves fresh weight; SPSt_FW, single plant stem fresh weight; StV, stem volume.

### Genome-wide correlation-based network analysis reveals large- and small-effect loci contributing to plant architectural and biomass yield traits

To identify highly connected SNPs beyond intra-chromosome limitations, we conducted a genome-wide, correlation-based network analysis. This involved performing partial correlation analysis among genotype calls for all possible SNP pairs, followed by projection of the correlation network onto Cytoscape (v3.10.1). The clustering coefficient algorithm in cytoHubba was used to determine the highest connectivity, using a correlation coefficient cutoff of *r=*0.231 (*P*<0.05). Biomass yield is a complex trait, predominantly influenced by both large- and numerous small-effect polymorphic loci (Mural *et al.*, 2021). Our analysis identified large-effect SNPs related to flowering (S04_39191115 and S04_19840847), plant height (*Dw2*, S07_52737620), and LLL (S06_42747863), as well as small-effect SNPs related to SPL_FW (S06_58127831), SPPani_FW (S01_13351970), TillN (S02_16386977), PlantN (S10_50508696), and LLL (S06_9288934) that showed the highest connections with other plant architectural and biomass yield traits (Fig. 4D).

## Discussion

Biomass yield is a complex trait comprising multiple plant architectural traits across various organs. It is typically regulated by numerous genes, many of which have relatively small effects, with environmental factors often playing a significant role (Miao *et al.*, 2019; Souza *et al.*, 2021). Sorghum, a member of the Andropogoneae tribe, stands out as a preferred bioenergy crop due to its diploid nature, extensive breeding history, substantial natural diversity, and small genome of 750 Mb. Identifying genomic regions associated with plant architectural and biomass yield traits not only facilitates the discovery of uncharacterized genes but also supports the design of bioenergy crops capable of adapting to changing climate conditions (Ain *et al.*, 2022).

We utilized the SAP, which primarily consists of grain sorghum selected to maximize genetic and phenotypic diversity (Casa *et al.*, 2008). The SAP includes temperate-adapted breeding lines and converted photoperiod-insensitive tropical accessions from the Sorghum Conversion Program (Klein *et al.*, 2008). In contrast, the BAP (biomass and sweet sorghum) consists of tall, photoperiod-sensitive, and late-maturing accessions (Brenton *et al.*, 2016). The average flowering time and plant height in the BAP were approximately 30 d longer and twice as high, respectively, compared with the SAP. This difference would have been even more pronounced if all photoperiod-sensitive accessions in the BAP had been included (Brenton *et al.*, 2016; Kumar *et al.*, 2024). Both SAP and BAP accessions may not exhibit trait segregation in a straightforward manner. However, in multi-parent populations (NAM or MAGIC), the trait of interest might segregate transgressively, showing a wider range of phenotypic variation than the parent lines (Bouchet *et al.*, 2017; Boatwright *et al.*, 2021). Therefore, if desirable phenotypic traits include multiple plant architectural and biomass yield traits, the BAP accessions or multi-parent populations may be less suitable for temperate environments. Instead, the SAP, which is better adapted to temperate conditions, was utilized to phenotype plant architectural and biomass yield traits.

Fourteen plant architectural and 10 corresponding biomass yield traits were phenotyped across the SAP panel over two growing seasons (Figs 1, 2A; Supplementary Table S2, S3). Phenotyping these traits at the population level presents several difficulties, primarily because most phenotyping was destructive and performed manually, making the process labor-intensive and time-consuming. This approach could affect the repeatability and heritability of the measurements. In our study, except for two biomass yield traits, namely ‘total plot fresh weight’ and ‘total plot dry weight’, all phenotyping was performed manually and involved mostly destructive processes. To capture the phenotypic diversity of each trait among a large number of SAP accessions, two plants per plot for plant architectural traits and one plant per plot for biomass yield traits were phenotyped in each growing season.

Both plant architectural and biomass yield trait phenotypic data showed significant variation across accessions, as indicated by their CV. The CV was highest for ‘tiller number per plant’ and lowest for ‘flowering’ over the two growing seasons, suggesting that the former trait is the least stable, with greater phenotypic dispersion relative to the mean, while the latter is the most stable within the panel (Fig. 1, 2A). Furthermore, substantial variation across two growing seasons notably influenced all traits, except ‘plant height up to flag leaf’, ‘single plant dry weight’, and ‘total plot dry weight’, as evidenced by the lowest percentage change across two growing seasons. Most of the plant architectural traits demonstrated higher heritability compared with biomass yield traits across growing seasons within the trial location. This may be due to the higher replication in measuring plant architectural traits compared with biomass yield traits. Additionally, the heritability of traits across different locations was not included in this analysis and requires further investigations to fully understand the environmental interaction affecting these traits. Taken together, the SAP demonstrates substantial phenotypic diversity in terms of both plant architecture and biomass yield traits, providing a robust foundation for GWAS analysis.

Recently Boatwright *et al.* (2022) generated high-density genomic marker sets for the SAP populations, including SNPs, indels, and copy number variants. This resource not only enhances the identification of various genetic variants, thereby improving genetic dissection and genome-wide prediction, but also advances pan-genomic efforts for future sorghum studies. In the present study, we aimed to expand our knowledge of the genes and genomic regions that regulate plant architectural and biomass yield traits in the SAP population. We performed GWAS analyses utilizing 569 305 high-density SNP markers obtained from Miao *et al.* (2020). Our GWAS identified a total of 321 significant SNPs associated with plant architectural and biomass yield traits across two growing seasons (Fig. 3; Supplementary Table S6). Biomass, being a complex trait, showed high polygenicity, predominantly influenced by numerous small-effect polymorphic loci. In our study, only 11.1% of significant SNPs had large effects (ranging from 10% to 38.4%) on the phenotypic traits, primarily impacting plant height and stem volume. The remaining 88.9% had small effects that ranged from 2.53% to 9.99% (Supplementary Table S6), which was consistent with previous findings (Habyarimana *et al.*, 2020).

Our GWAS analyses identified at least three previously known flowering time regulators (*Ma1*, *Ma2*, and *Ma6*) and three loci regulating plant height (*Dw1*, *Dw2*, and *Dw3)*. The *Dw1* locus exhibited a conserved, large-effect pleiotropic role, regulating not only plant height but also stem volume and stem biomass yield traits (Breitzman *et al.*, 2019; Mural *et al.*, 2021). We also observed strong linkage between *Ma1* and *Dw2* loci on chromosome 6, both exerting a significant effect on sorghum flowering, final height, and several biomass yield traits (Supplementary Fig. S14). During backcrossing and the sorghum conversion process, both loci were often introgressed together to achieve early maturity and short stature, facilitating the adaptation of tropical sorghum germplasm to temperate latitudes (Quinby and Karper, 1954; Higgins *et al.*, 2014). Additionally, our GWAS analyses identified new, uncharacterized regulators of plant architectural traits (plant height, stem, leaf, panicle, and tiller) and corresponding biomass yield traits. These regulators were identified based on SNP effects, PVE, and SNPs located within their genomic regions, although no detailed functional validation has been performed in sorghum to date. However, homologous genes in other plant species have shown significant impacts on plant architectural traits (Irish *et al.*, 1994; Nogueira *et al.*, 2007; Zhu *et al.*, 2010).

Our study also identified various primary and secondary metabolic pathways known to regulate sorghum biomass. For nearly all groups of plant architectural and biomass yield traits, several significantly associated SNPs were close to genes involved in carbohydrate metabolism, structural polysaccharide biosynthesis, lipid biosynthesis, flavonoids, and lignin biosynthesis. Altered expression of these genes contributes to the production of leaf (photosynthate), stem (sugar), grain (starch), and stem cellulosic and secondary cell wall (glucan, xylan, lignin) biomass in sorghum (Turner *et al.*, 2016). Phytohormones also play a crucial role in regulating plant height and leaf/stem/tiller traits (Borrell *et al.*, 2000; Grant *et al.*, 2023). For instance, plant height loci *Dw1* and *Dw3* co-localized with genes involved in brassinosteroid signaling and auxin efflux transporter, respectively (Grant *et al.*, 2023). Similarly, several SNPs related to leaf traits were located near signaling or biosynthetic genes related to auxin [*YUC flavin monooxygenase*, *auxin response factor* (*ARF*) *14*], brassinosteroids (*Sterol desaturase*), cytokinin (*cytokinin dehydrogenase*: SNP within genomic sequence), and jasmonic acids (*jasmonate ZIM domain-containing protein*, *lipoxygenase*). Recent research by Qiao *et al.* (2022) in rice demonstrated that auxins (*OsARF4*) participate in brassinosteroid signaling to regulate leaf architecture, while cytokinins delay senescence, and jasmonic acids confer resistance to biotic stresses (Barna, 2022; Hewedy *et al*., 2023).

Our GWAS analyses identified several transcription factors, including TCP, PLANT A/T-RICH SEQUENCE- AND ZINC-BINDING PROTEIN, MYBs, NAC, WRKYs, AP2, and Homeodomain, which are known to regulate biomass in sorghum. Previously, Hennet *et al.* (2020) identified MYB and NAC as master regulators involved in cell wall regulation. Overexpression of *SbMyb60* impacts both primary (cell wall polysaccharide) and secondary metabolism (lignin) (Scully *et al.*, 2016). The synergistic action of GROWTH-REGULATING FACTOR (GRF) and its transcription cofactor GRF-INTERACTING FACTOR 1 (GIF1), influences various developmental processes in plants, including leaf size and longevity (Debernardi *et al.*, 2014). Our analysis also identified *SbGRF6*, which co-localized with a SNP associated with leaf traits. This protein showed 53% identity with wheat *TaGRF4*. Previous work by Debernardi *et al.* (2020) showed that a fusion protein combining *TaGRF4–GIF* substantially increases the efficiency and speed of regeneration in wheat. Recently, Li *et al.* (2024) established an efficient sorghum transformation strategy using the GRF4–GIF1/ternary vector system, which could be a tool for studying gene function and trait improvement in sorghum.

Correlation analysis between plant architectural and biomass yield traits across two growing seasons revealed consistent patterns, indicating the stability of these traits (Fig. 2B; Supplementary Fig. S4). A positive correlation between plant architectural and biomass yield traits likely arises because many biomass yield traits in our study derive from individual plant parts that contribute to overall plant architecture. At the genomic level, substantial overlap in genomic regions and pleiotropic loci suggests a shared genetic mechanism underlying both plant architectural and biomass yield traits (Fig. 4A; Supplementary Fig. S12; Supplementary Tables S6, S13) (Mural *et al.*, 2021). In our study, GR_121 co-localized with SNPs related to plant height (*Dw2*), stem volume, largest leaf length, and flowering. The flowering locus has enhanced photoperiod sensitivity and delays flowering (Murphy *et al.*, 2014), while other SNPs in the same genomic region has been linked to maximized overall plant growth, as evidenced by increased plant height, which significantly increases leaf and stem biomass yield (Murphy *et al.*, 2011). GR_145 was associated with a plant height regulating locus, *Dw3*, which, together with TCP, cyclins, and other genes, contributed to plant height, as well as leaf- and panicle-related traits. GR_178 co-localized with genes involved in photosynthesis and related metabolic processes, and the brassinosteroid signaling gene *Dw1*, which regulates height and biomass in sorghum (Hirano *et al.*, 2017). In contrast, some plant architectural traits, including tiller number per plant and plant number, exhibited a negative correlation with all the biomass yield traits (Fig. 2B; Supplementary Fig. S4). This observation has been substantiated at the genetic level, where SNPs related to these traits showed trade-off with biomass yield traits (Supplementary Figs S15, S16), indicating competition and antagonistic selection with biomass traits (Yang *et al.*, 2019; Burgess and Cardoso, 2023).

The ability to identify both large- and small-effect loci associated with a phenotype of interest is paramount for breeders. Network analysis highlighted SNPs with both larger and smaller effects on flowering, plant height (*Dw2*), leaf-related traits, plant number per m^2^, and tiller number per plant. These loci act as major contributors and exhibit connections with other genetic loci related to plant architecture and biomass yield traits (Fig. 4D). Understanding these loci is essential for gaining a comprehensive understanding of the genetic regulation of complex traits of economic importance, such as biomass. They can be effectively utilized in developing specialized plant feedstocks for bioenergy in sorghum.

The diversity among the SAP population accessions can be leveraged to enhance plant architecture and biomass yield traits. Our detailed phenotyping has allowed us to identify several parent lines with potential as donors for biomass traits (Supplementary Fig. S3). Promising parent lines for improving specific traits include those with higher values for plant height (PI 656038 and PI 651496), flowering (PI 656017), leaf biomass (PI 656107), and stem biomass (PI 656023). These accessions exhibit favorable phenotypic characteristics that contribute to increased biomass yield.

Our work offers insight into the genetic basis of various plant architectural and biomass yield traits in sorghum. We observed natural variation in these traits and identified both well-characterized and uncharacterized loci, which serve as a foundation for further functional investigation. Additionally, we identified conserved pleiotropic loci, shared genomic regions, highly correlated SNP blocks, and highly connected SNPs influencing multiple plant architectural and biomass yield traits. In particular, loci and significant SNP markers related to plant height, flowering, and leaf-related traits are of great interest. The strategic combination of favorable alleles related to these traits through pyramiding will be beneficial for designing bioenergy sorghum crops.

## Supplementary data

The following supplementary data are available at *JXB* online.

Fig. S1. Graphical representation of average monthly temperature (°C) and average monthly precipitation (cm) over two growing seasons of sorghum at the Michigan State University research farm.

Fig. S2. Spatial distribution of raw data, fitted data, and regression analysis between raw and fitted data for days to 50% flowering and plant height up to the flag leaf.

Fig. S3. Principal component analysis of phenotypic data related to plant architectural and biomass yield traits over two growing seasons (2020 and 2021).

Fig. S4. Pearson correlation analysis between plant architectural and biomass yield traits in the 2021 growing season.

Fig. S5. Genome-wide association study (GWAS) for plant architectural and biomass yield traits in the Sorghum Association Panel across two growing seasons.

Fig. S6. Summary of SNPs identified from genome-wide association studies for plant architectural and biomass yield traits in the Sorghum Association Panel across two growing seasons.

Fig. S7. Gene ontology (GO) enrichment analysis of genes identified around 321 SNPs.

Fig. S8. Allelic variation of SNPs associated with *Ma6* and *Ma1* loci and their impact on flowering.

Fig. S9. Allelic variation of a SNP associated with a TCP transcription factor and its impact on leaf biomass (SPL_FW and SPL_DW).

Fig. S10. Allelic variation of the locus near PLANT A/T-RICH Sequence-and zinc-binding protein and its impact on leaf biomass (SPL_FW and SPL_DW).

Fig. S11. Allelic variation of the locus near sorghum GROWTH-REGULATING FACTOR (*SbGRF*) and its impact on leaf biomass (SPL_FW and SPL_DW).

Fig. S12. Distribution of significant SNPs across various genomic regions.

Fig. S13. Chromosome-wise correlation analysis among SNPs.

Fig. S14. Allelic variation of SNPs related to *Ma1* and *Dw2* loci and their impact on multiple biomass yield traits.

Fig. S15. Allelic variation of the locus associated with PlantN and its impact on plant biomass (SP_FW and SP_DW).

Fig. S16. Allelic variation of the locus associated with TillN and its impact on plant biomass (SP_FW and SP_DW).

Table S1. List of sorghum accession from the Sorghum Association Panel and their race classifications used in this study.

Table S2. Phenotypic data of plant architectural traits.

Table S3. Phenotypic data of plant biomass yield traits.

Table S4. Statistical analysis of phenotypic data for symmetrical distribution, positive and negative skewness.

Table S5. Two-way analysis of variance (ANOVA) of plant architectural and biomass yield traits from the sorghum association panel across two growing seasons (2020 and 2021).

Table S6. List of significant SNPs identified from genome-wide association studies for plant architectural and biomass yield traits in the Sorghum Association Panel across two growing seasons.

Table S7. List of genes in the vicinity of significant SNPs.

Table S8. List of genes associated with significant SNPs related to flowering.

Table S9. List of genes identified around significant SNPs related to leaf traits and linked biomass yield traits.

Table S10. List of genes identified around significant SNPs related to plant height, stem-related traits, and biomass yield traits.

Table S11. List of genes identified around significant SNPs related to plant density, tiller number per plant, and total plot biomass yield traits.

Table S12. List of genes identified around significant SNPs related to panicle traits and corresponding biomass yield traits.

Table S13. List of genomic regions associated with 321 significant SNPs mapped to sorghum chromosomes in 600 kb interval.

eraf012_suppl_Supplementary_Tables_S1-S13

eraf012_suppl_Supplementary_Figures_S1-S16

## Data Availability

All datasets will be available upon request.
