# Supplementary material for: Unveiling shared genetic regulators of plant architectural and biomass yield traits in the Sorghum Association Panel
Source: J Exp Bot. 2025 Jan 11;76(6):1625–43. doi: 10.1093/jxb/eraf012 (PMC11981901; doi:10.1093/jxb/eraf012)
Supplement: eraf012_suppl_Supplementary_Figures_S1-S16 [file eraf012_suppl_supplementary_figures_s1-s16.pdf]

Supplementary data

Unveiling shared genetic regulators for plant architectural and biomass yield traits in sorghum

Supplementary Figures: 16

Abbreviations used in this file were as follows:

- Final green leaves (FGL)
- Largest leaf number from top (LLN_top)
- Largest leaf length (LLL)
- Largest leaf width (LLW)
- Largest leaf area (LLA)
- Plant height up to flag leaf (PF_FL)
- Plant height up to panicle (PH_Pani)
- Stem diameter (StD)
- Stem volume (StV)
- Plant number per square meter (PlantN)
- Panicle number per square meter (PaniN)
- Panicle length (PaniL)
- Tiller number per plant (TillN)
- Single plant_fresh weight and dry weight (SP_FW and SP_DW)
- Single plant 'stem'_fresh weight and dry weight (SPSt_FW and SPSt_DW)
- Single plant 'leaves'_fresh weight and dry weight (SPL_FW and SPL_DW)
- Single plant 'panicle'_fresh weight and dry weight (SPPani_FW and SPPani_DW)
- Total 'plot' fresh weight and dry weight (TPlot_FW and TPlot_DW).

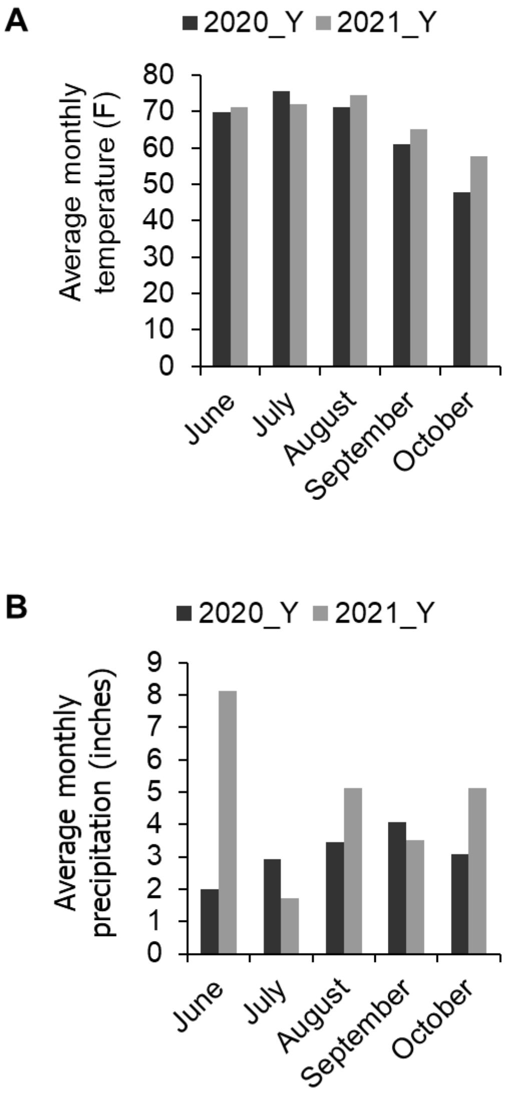

Fig. S1 (A-B). Graphical representation of average monthly temperature (°F) and average monthly precipitation (inches) over two growing seasons of sorghum at the Michigan State University research farm. The data was obtained from this site. https://www.wunderground.com/history/monthly/us/mi/lansing/KLAN/date/2022-9.

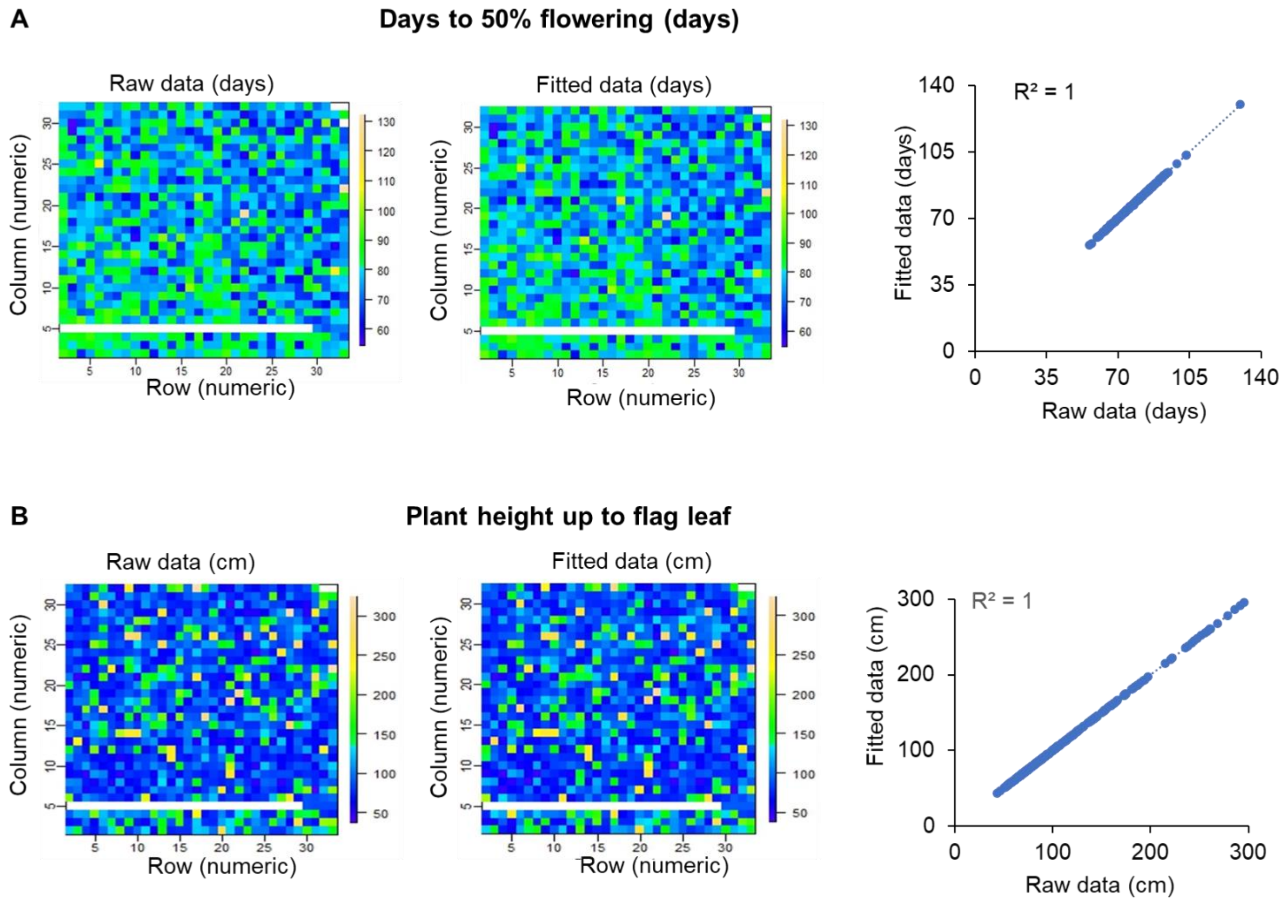

Fig. S2 (A-B). Spatial distribution of raw data, fitted data, and regression analysis between raw and fitted data for days to 50% flowering (A) and plant height up to the flag leaf (B). Spatial analysis was conducted using the SpATS model, with data plotted against row and column positions.

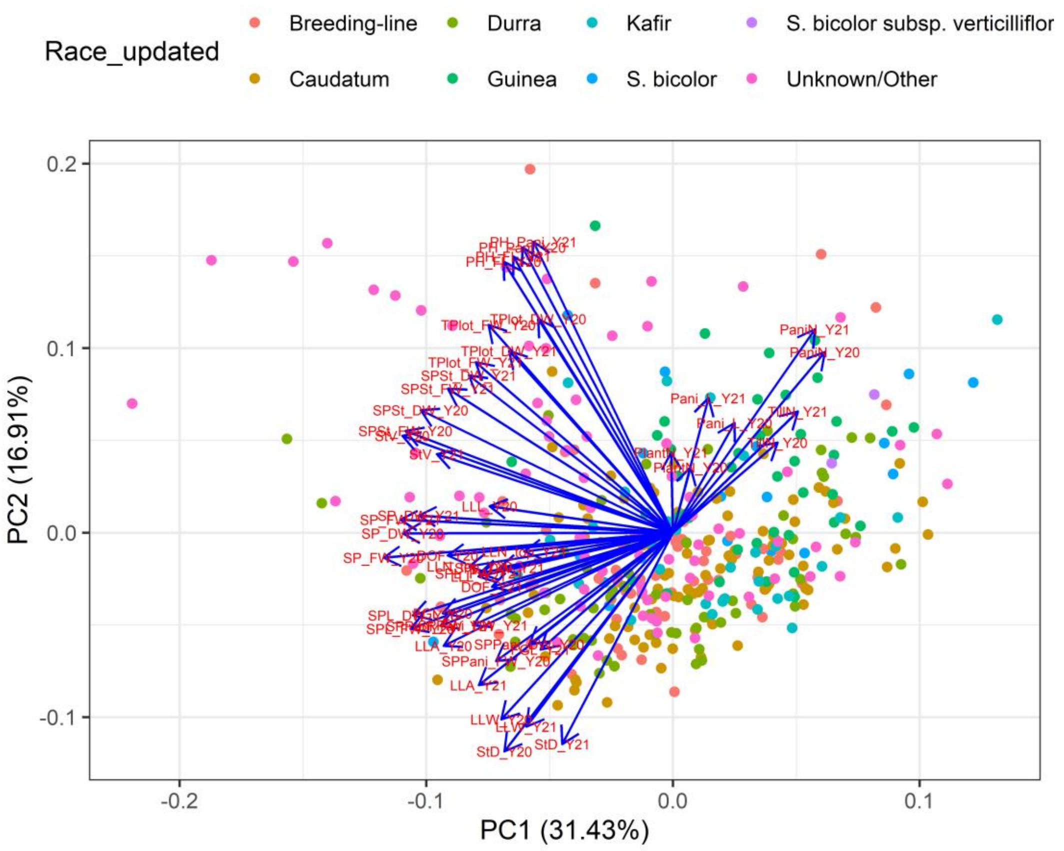

Fig. S3. Principal component analysis of phenotypic data related to plant architectural and biomass yield traits over two growing seasons (2020 and 2021). The phenotypic data were collected from the Sorghum Association Panel, which include different botanical races.

Biomass yield trait

Colour scale: -1 | -0.5 | 0 | 0.5 | 1

Architectural trait	SP_FW	SP_DW	SPSt_FW	SPSt_DW	SPL_FW	SPL_DW	SPPani_FW	SPPani_DW	TPlot_FW	TPplot_DW
StV	0.55	0.50	0.52	0.46	0.27	0.20	0.45	0.38	0.51	0.45
PH_FL	0.35	0.36	0.57	0.57	0.05 ns	0.03 ns	0.12	0.11	0.64	0.66
PH_Pani	0.31	0.31	0.54	0.55	0.01 ns	-0.01 ns	0.07 ns	0.06 ns	0.63	0.64
StD	0.25	0.19	-0.03 ns	-0.11	0.27	0.21	0.41	0.34	-0.11	-0.18
Flowering	0.41	0.27	0.25	0.13	0.44	0.33	0.38	0.24	0.33	0.15
FGL	0.30	0.17	0.14	0.03 ns	0.36	0.27	0.30	0.18	0.18	0.03 ns
LLN_top	0.33	0.24	0.29	0.21	0.36	0.28	0.24	0.14	0.32	0.16
LLA	0.41	0.35	0.12	0.05 ns	0.33	0.27	0.53	0.46	0.21	0.14
LLL	0.35	0.31	0.20	0.13	0.26	0.21	0.39	0.34	0.36	0.31
LLW	0.34	0.29	0.03 ns	-0.03 ns	0.28	0.22	0.50	0.44	0.05 ns	-0.01 ns
PlantN	-0.24	-0.29	-0.04 ns	-0.06 ns	-0.14	-0.17	-0.34	-0.38	0.28	0.26
PaniN	-0.31	-0.30	-0.01 ns	0.04 ns	-0.15	-0.10	-0.50	-0.49	0.03 ns	0.05 ns
TillN	-0.11	-0.07 ns	0.00 ns	0.06 ns	-0.04 ns	0.01 ns	-0.19	-0.16	-0.17	-0.14
Pani_L	-0.08 ns	-0.10	0.05 ns	0.05 ns	-0.12	-0.14	-0.16	-0.17	0.13	0.12

Fig. S4. Pearson correlation analysis between plant architectural and biomass yield traits in the 2021 growing season. Positive correlations are indicated in red, while negative correlations are shown in blue. All correlations, whether positive or negative, were considered significant at $p \leq 0.05$, with non-significant correlations denoted as 'ns'.

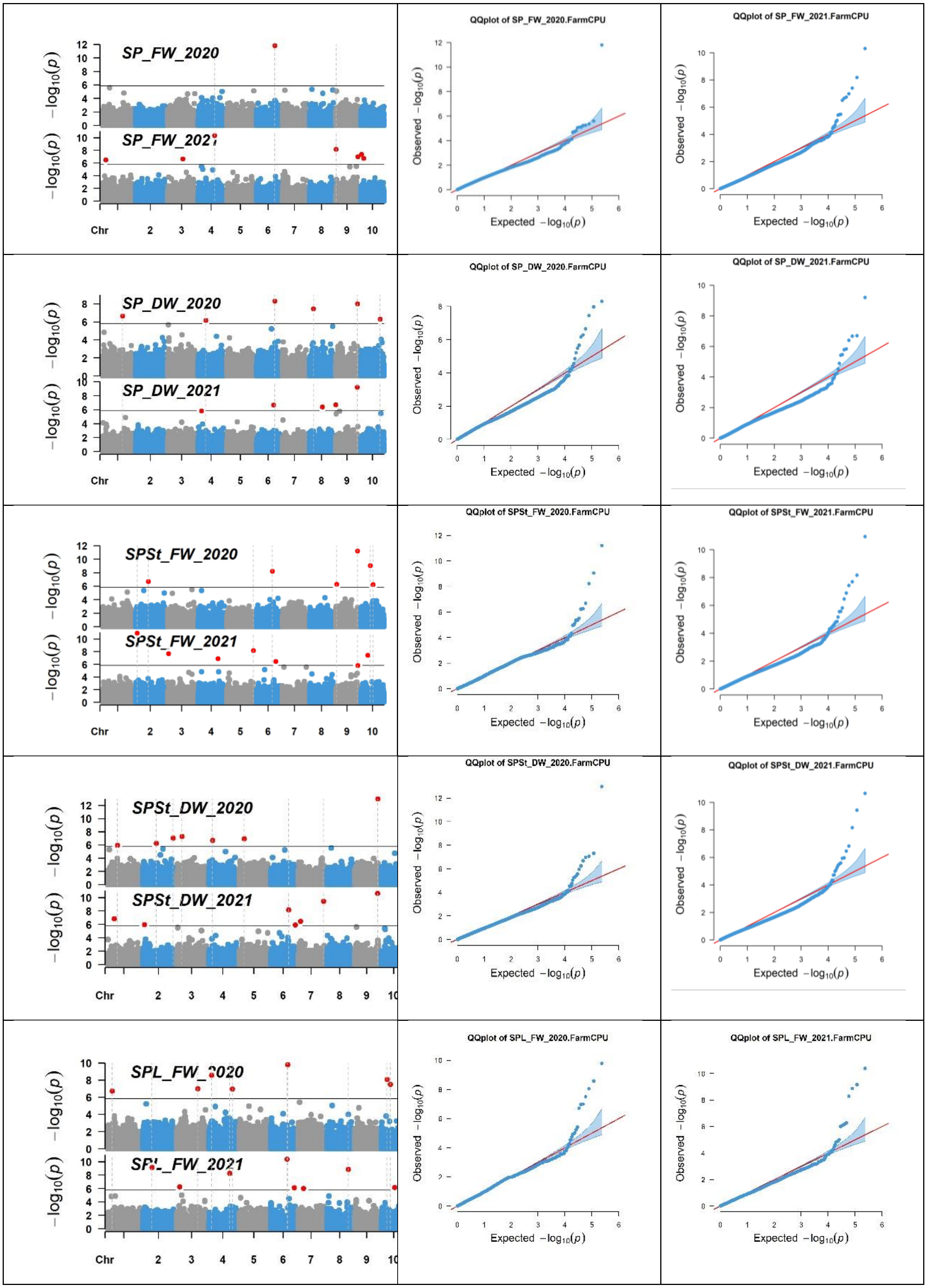
SP_FW_2020
SP_FW_2021
QQplot of SP_FW_2020.FarmCPU
QQplot of SP_FW_2021.FarmCPU
SP_DW_2020
SP_DW_2021
QQplot of SP_DW_2020.FarmCPU
QQplot of SP_DW_2021.FarmCPU
SPSt_FW_2020
SPSt_FW_2021
QQplot of SPSt_FW_2020.FarmCPU
QQplot of SPSt_FW_2021.FarmCPU
SPSt_DW_2020
SPSt_DW_2021
QQplot of SPSt_DW_2020.FarmCPU
QQplot of SPSt_DW_2021.FarmCPU
SPL_FW_2020
SPL_FW_2021
QQplot of SPL_FW_2020.FarmCPU
QQplot of SPL_FW_2021.FarmCPU
Chr
−log10(p)
Observed −log10(p)
Expected −log10(p)

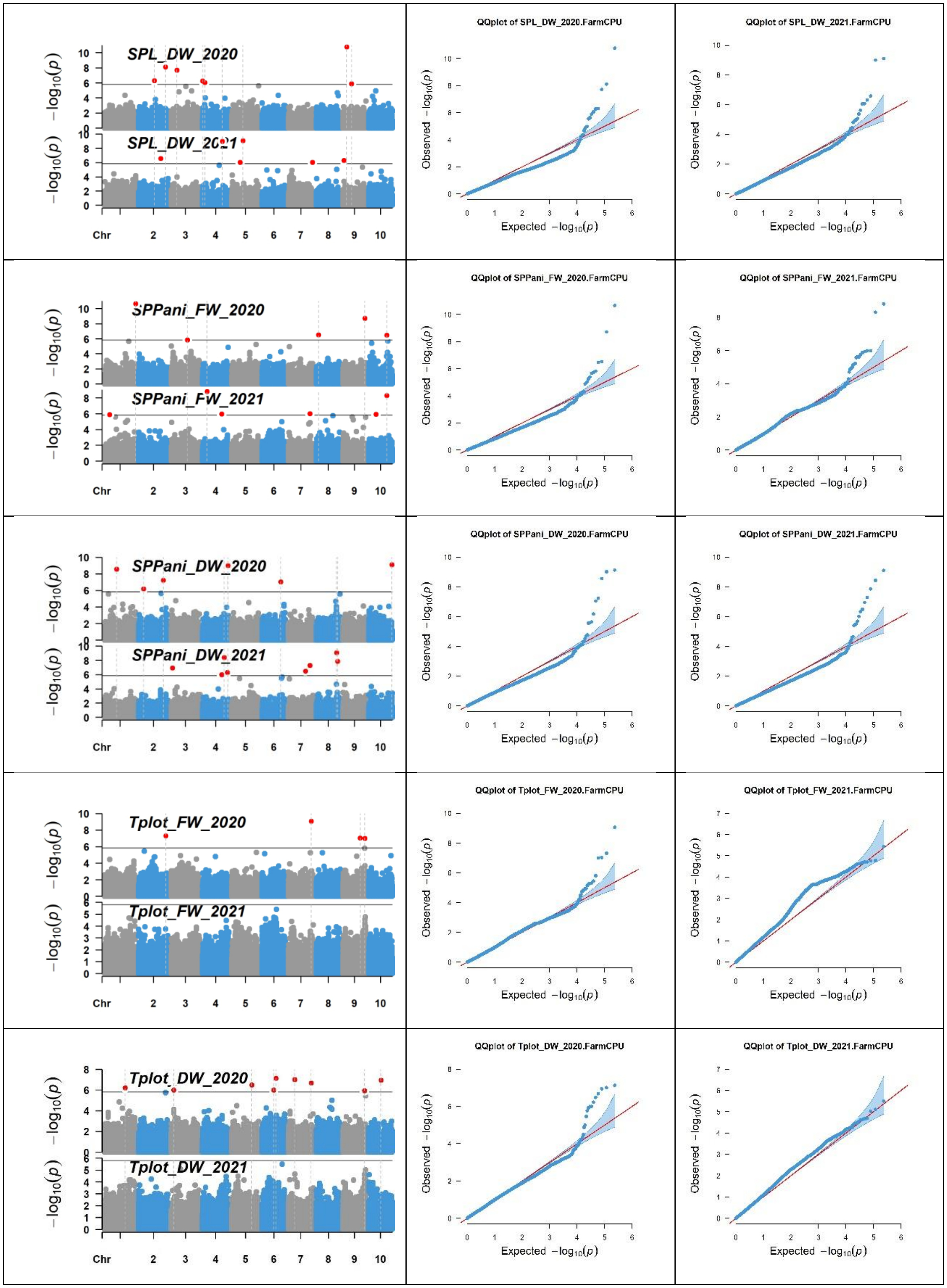
SPL_DW_2020
SPL_DW_2021
SPPani_FW_2020
SPPani_FW_2021
SPPani_DW_2020
SPPani_DW_2021
Tplot_FW_2020
Tplot_FW_2021
Tplot_DW_2020
Tplot_DW_2021
QQplot of SPL_DW_2020.FarmCPU
QQplot of SPL_DW_2021.FarmCPU
QQplot of SPPani_FW_2020.FarmCPU
QQplot of SPPani_FW_2021.FarmCPU
QQplot of SPPani_DW_2020.FarmCPU
QQplot of SPPani_DW_2021.FarmCPU
QQplot of Tplot_FW_2020.FarmCPU
QQplot of Tplot_FW_2021.FarmCPU
QQplot of Tplot_DW_2020.FarmCPU
QQplot of Tplot_DW_2021.FarmCPU
$-\log_{10}(p)$
Chr
Observed $-\log_{10}(p)$
Expected $-\log_{10}(p)$

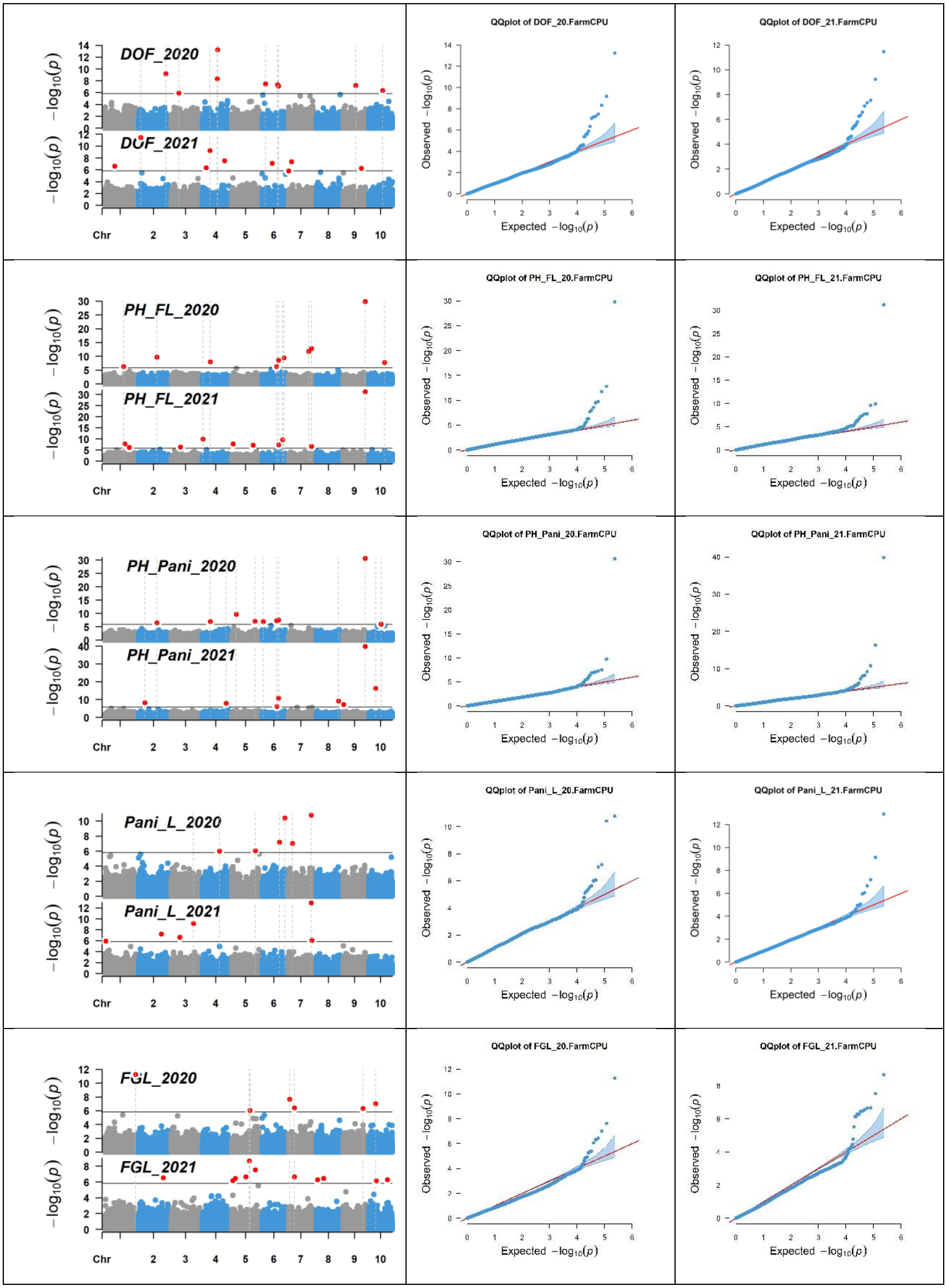

DOF_2020
DOF_2021
QQplot of DOF_20.FarmCPU
QQplot of DOF_21.FarmCPU
PH_FL_2020
PH_FL_2021
QQplot of PH_FL_20.FarmCPU
QQplot of PH_FL_21.FarmCPU
PH_Pani_2020
PH_Pani_2021
QQplot of PH_Pani_20.FarmCPU
QQplot of PH_Pani_21.FarmCPU
Pani_L_2020
Pani_L_2021
QQplot of Pani_L_20.FarmCPU
QQplot of Pani_L_21.FarmCPU
FGL_2020
FGL_2021
QQplot of FGL_20.FarmCPU
QQplot of FGL_21.FarmCPU
$-\log_{10}(p)$
Chr
Observed $-\log_{10}(p)$
Expected $-\log_{10}(p)$

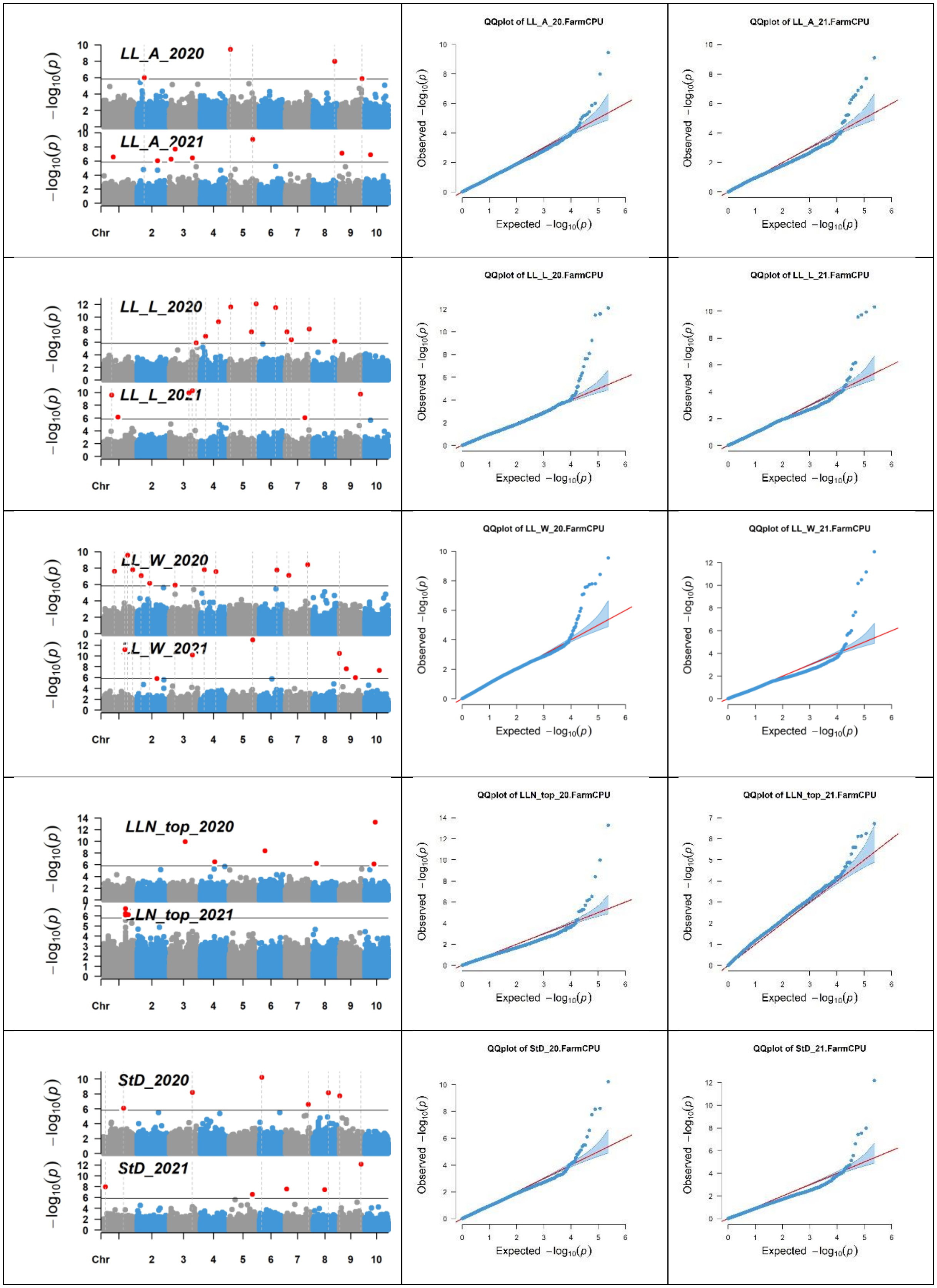

LL_A_2020
LL_A_2021
QQplot of LL_A_20.FarmCPU
QQplot of LL_A_21.FarmCPU
LL_L_2020
LL_L_2021
QQplot of LL_L_20.FarmCPU
QQplot of LL_L_21.FarmCPU
LL_W_2020
LL_W_2021
QQplot of LL_W_20.FarmCPU
QQplot of LL_W_21.FarmCPU
LLN_top_2020
LLN_top_2021
QQplot of LLN_top_20.FarmCPU
QQplot of LLN_top_21.FarmCPU
StD_2020
StD_2021
QQplot of StD_20.FarmCPU
QQplot of StD_21.FarmCPU
$-\log_{10}(p)$
Chr
Observed $-\log_{10}(p)$
Expected $-\log_{10}(p)$

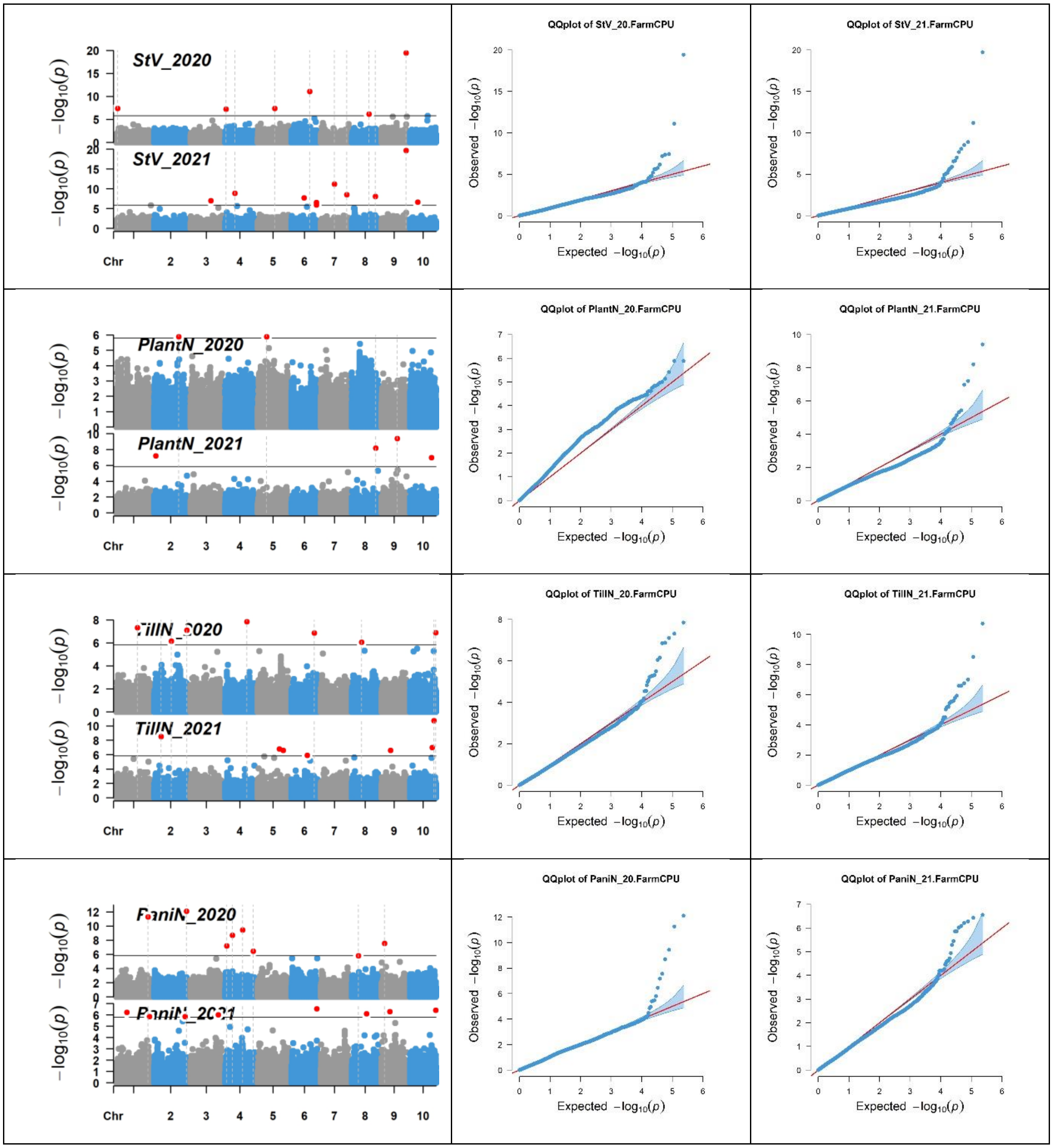

Fig. S5. Genome-wide association study (GWAS) for plant architectural and biomass yield traits in the Sorghum Association Panel across two growing seasons. Significant SNPs are visualized using Manhattan plot (left) and the quantile-quantile (Q-Q) plots (right). The horizontal grey line in the Manhattan plot indicates the significant threshold ($p \leq 2.4 \times 10^{-6}$).

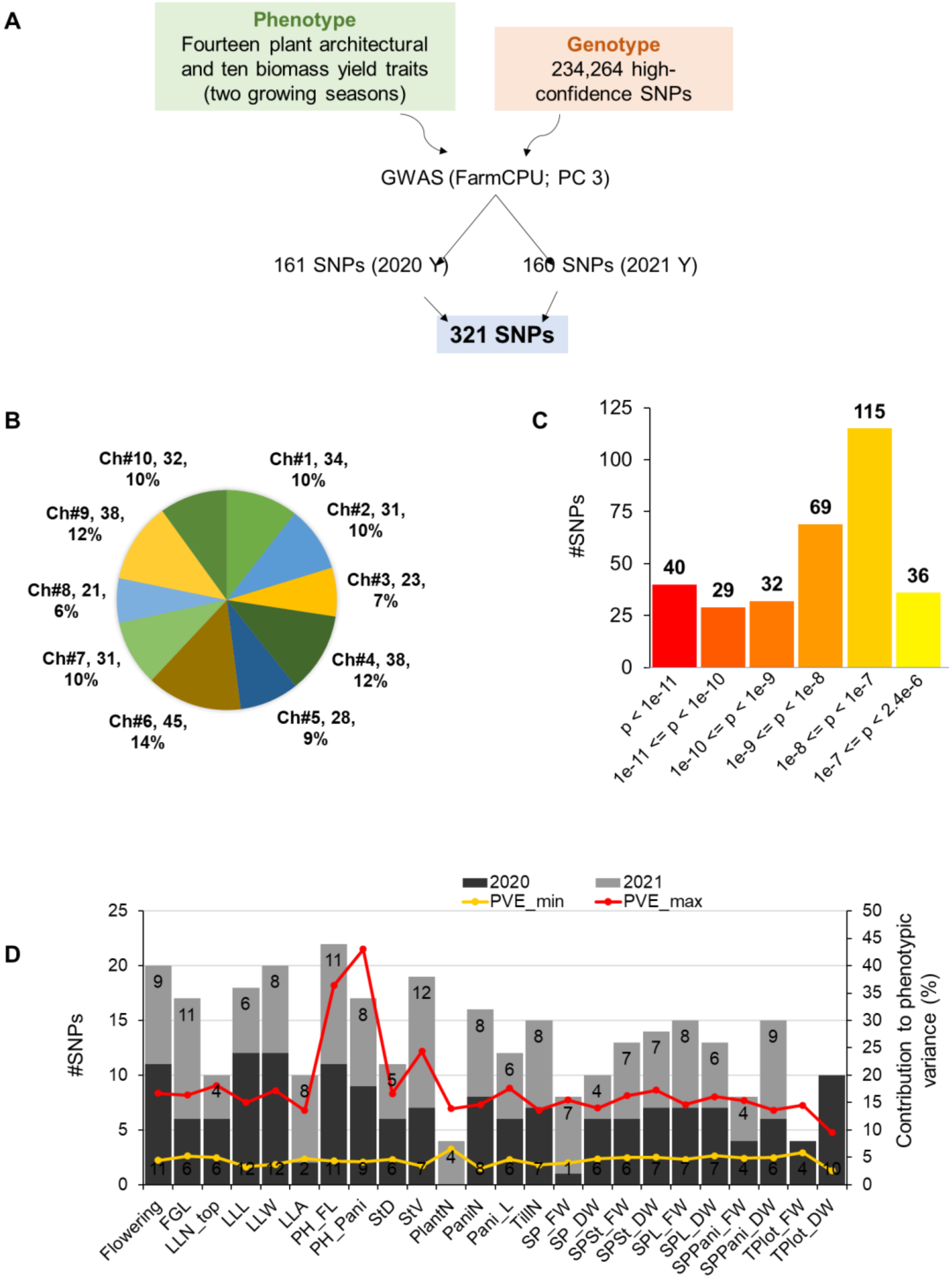

Fig. S6. Summary of SNPs identified from genome-wide association studies for plant architectural and biomass yield traits in the Sorghum Association Panel across two growing seasons.

A. Total number of SNPs identified using the FarmCPU-based GWAS method.

B. Chromosome-wise SNP distribution.

C. SNP partitioning based on $-\log_{10}(p\text{-values})$.

D. Trait-wise SNP distribution over two growing seasons. The plot also shows the percentage of phenotypic variance explained by small- and large-effect SNPs for each trait, including the minimum (PVE_min) and maximum (PVE_max) values.

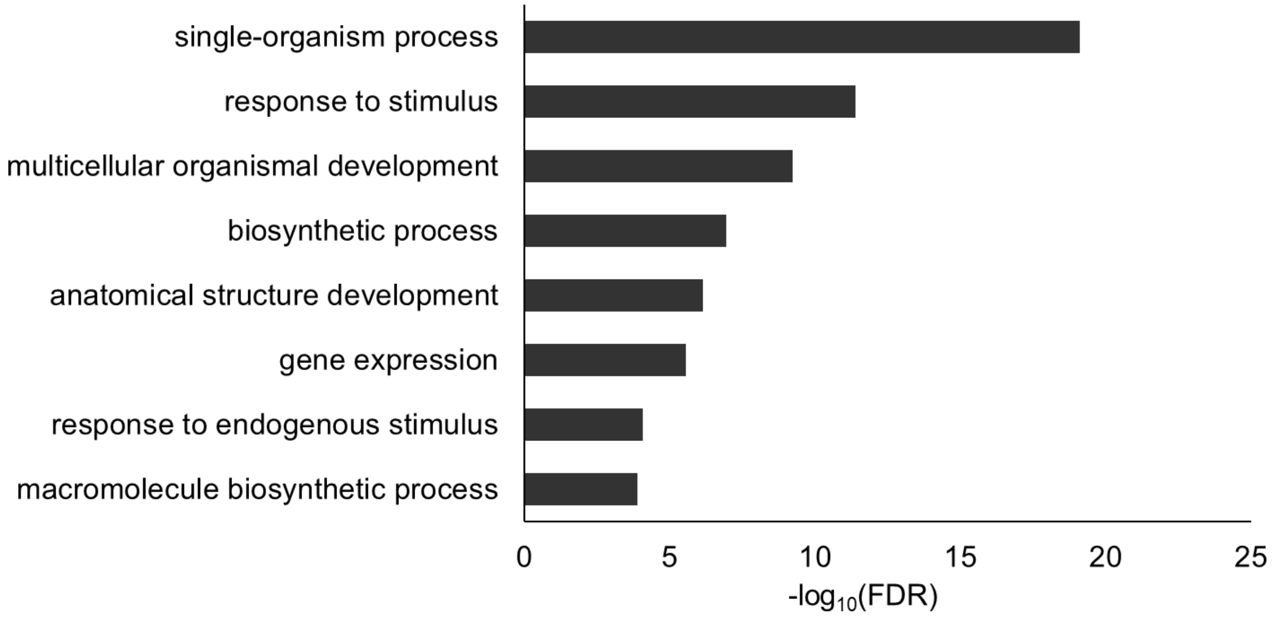

Fig. S7. Gene ontology (GO) enrichment analysis of genes identified around 321 SNPs. Approximately 2,700 genes were identified within a 150 kb range centered on each significant SNP. The list of these genes is provided in Supplementary Table S7.

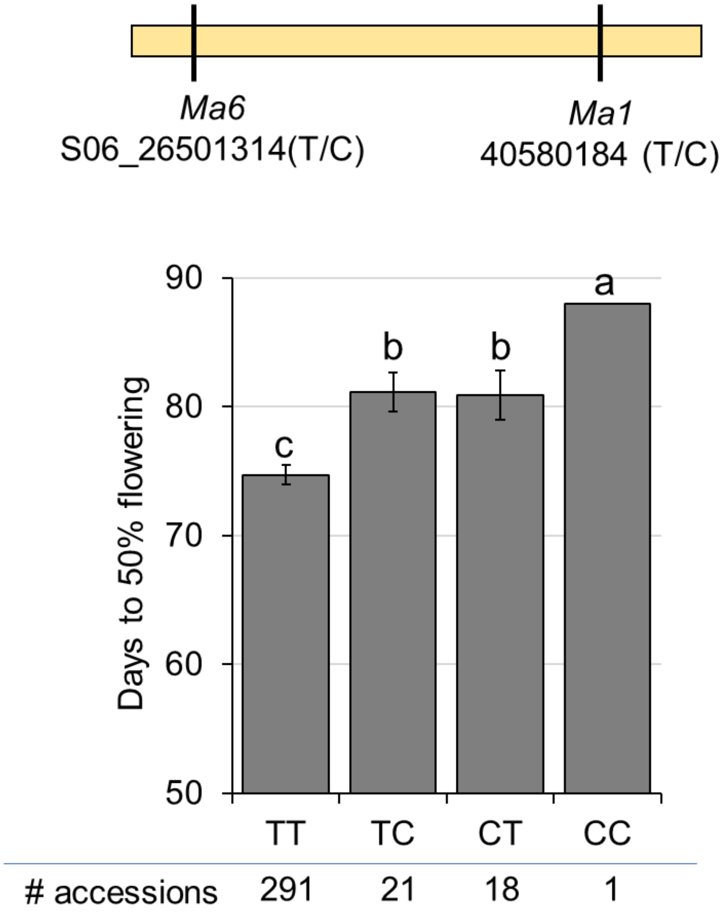

Fig. S8. Allelic variation of SNPs associated with *Ma6* and *Ma1* loci and their impact on flowering. Phenotypic data were obtained from the 2020 growing season. Data represents the mean and standard error of the mean for accessions carrying different allele combinations. Different letters above the bars indicate significant differences among accessions, as determined by one-way ANOVA with Tukey's HSD (post-hoc) test at $p \leq 0.05$.

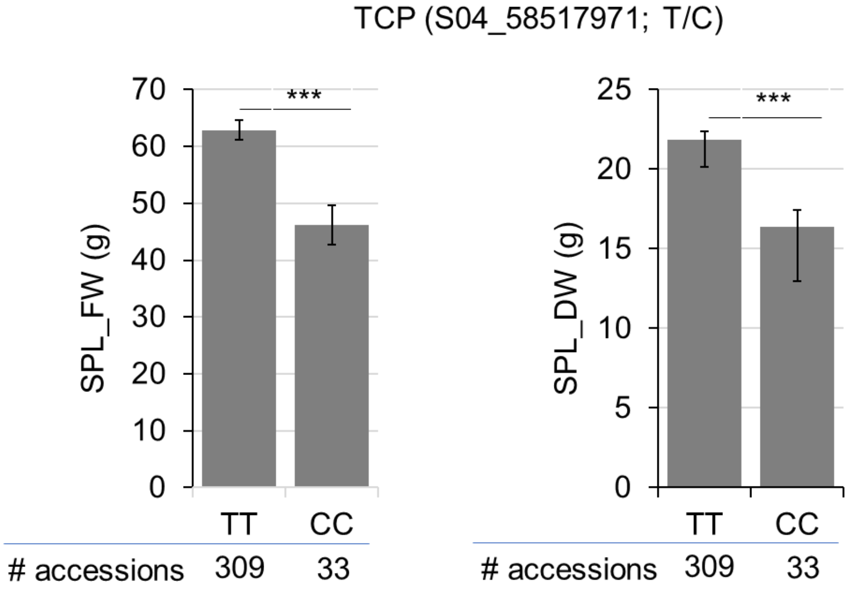

Fig. S9. Allelic variation of a SNP associated with a TCP transcription factor and its impact on leaf biomass (SPL_FW and SPL_DW). Phenotypic data were obtained from the 2020 growing season. Data represents the mean and standard error of the mean for accessions carrying different alleles. A Student's t-test was performed to assess significant differences between groups ($p < 0.05$*, $p < 0.01$**, $p < 0.001$***, *ns*: 'not significant').

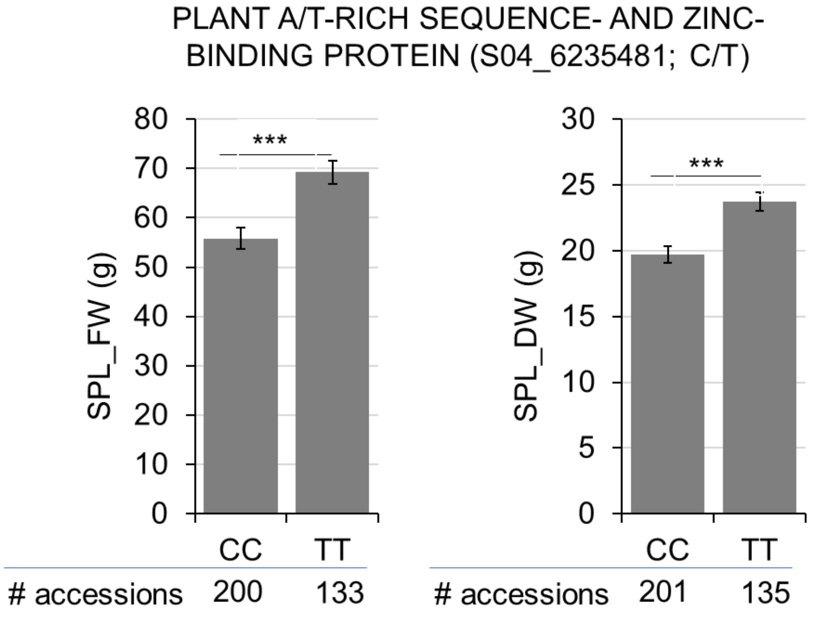

Fig. S10. Allelic variation of the locus near PLANT A/T-RICH Sequence-and zinc-binding protein and its impact on leaf biomass (SPL_FW and SPL_DW). Phenotypic data were obtained from the 2020 growing season. Data represents the mean and standard error of the mean for accessions carrying different alleles. A Student's t-test was performed to assess significant differences between groups ($p < 0.05$*, $p < 0.01$**, $p < 0.001$***, *ns*: 'not significant').

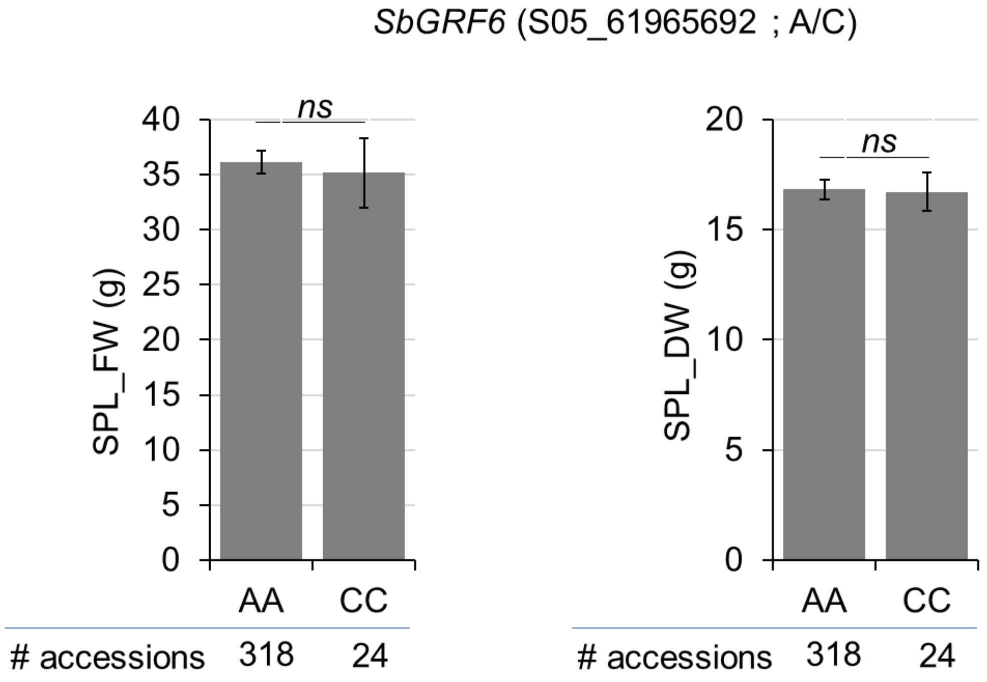

Fig. S11. Allelic variation of the locus near sorghum GROWTH-REGULATING FACTOR (*SbGRF*) and its impact on leaf biomass (SPL_FW and SPL_DW). Phenotypic data were obtained from the 2021 growing season. Data represents the mean and standard error of the mean for accessions carrying different alleles. A Student's t-test was performed to assess significant differences between groups ($p < 0.05$*, $p < 0.01$**, $p < 0.001$***, *ns*: 'not significant').

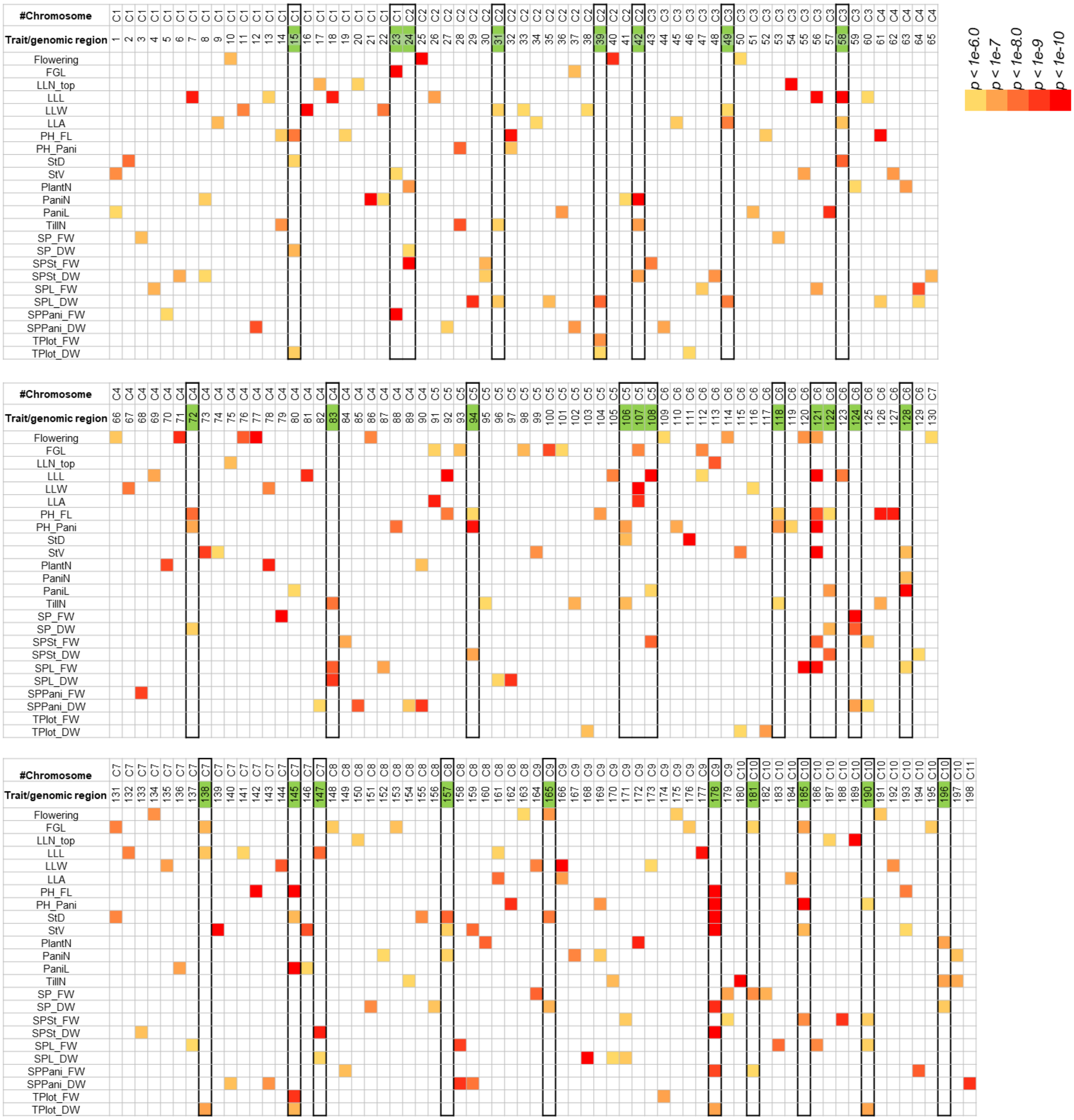

Fig. S12. Distribution of significant SNPs across various genomic regions. These regions are numbered 1 to 198, distributed across all 10 sorghum chromosomes. The significance of each SNP is color-coded based on the *p*-value of the association, with white boxes indicating no significant association. Solid boxes with green highlighting around specific genomic regions indicate hotspot areas associated with three or more traits, including plant architecture, biomass, or both. Further details of these genomic regions are provided in Supplementary Table S13.

Chromosome 1

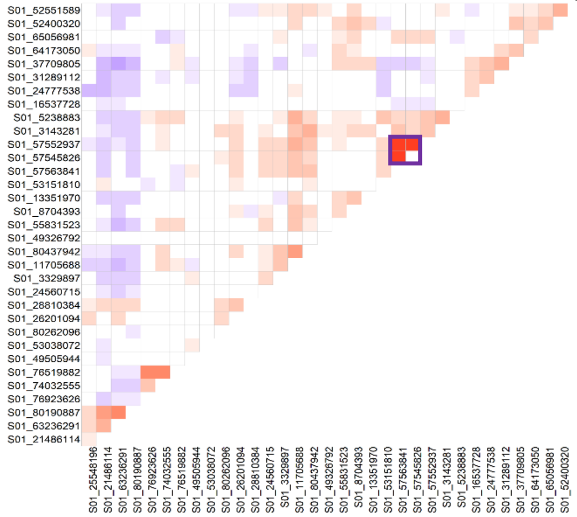

Chromosome 2

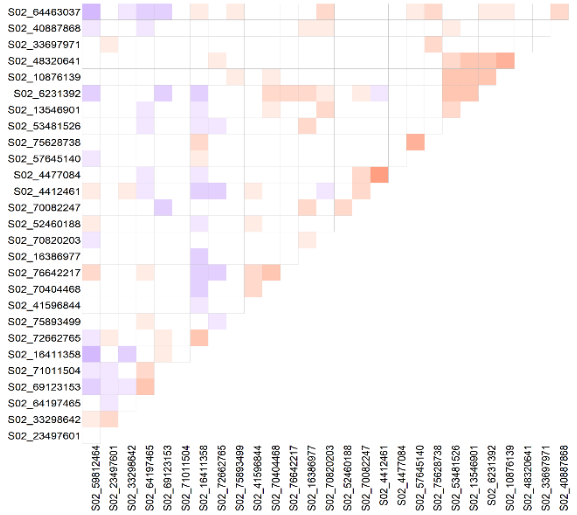

Chromosome 3

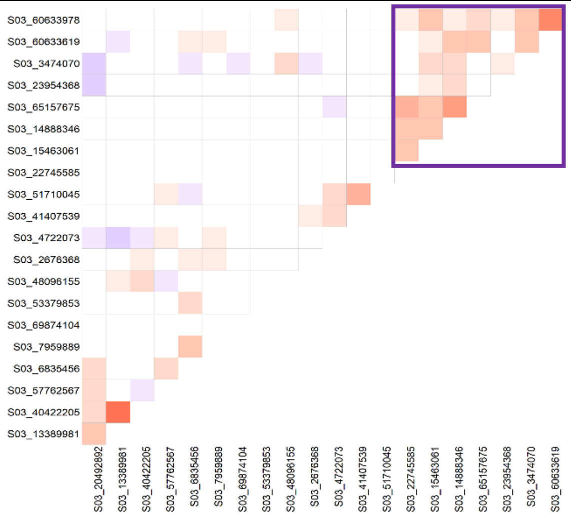

Chromosome 4

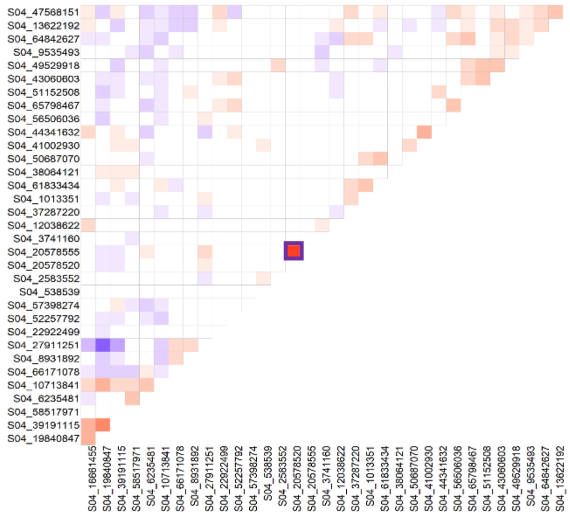

Chromosome 5

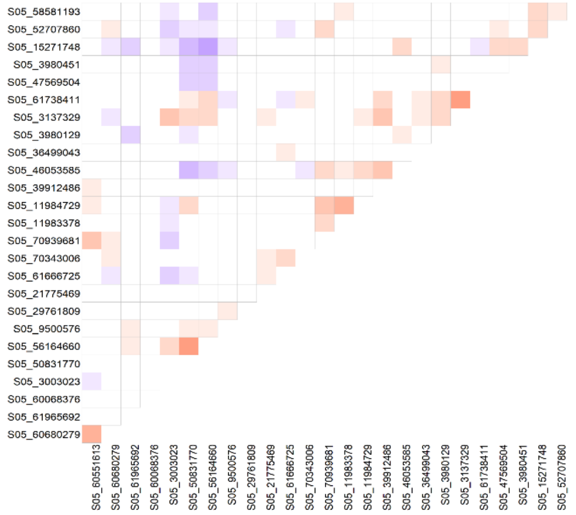

Chromosome 6

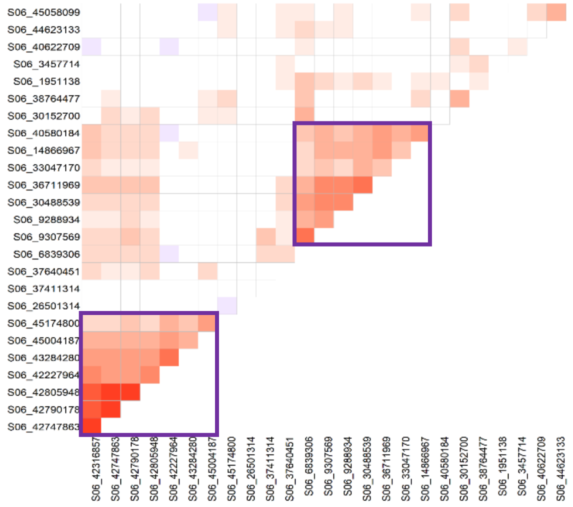

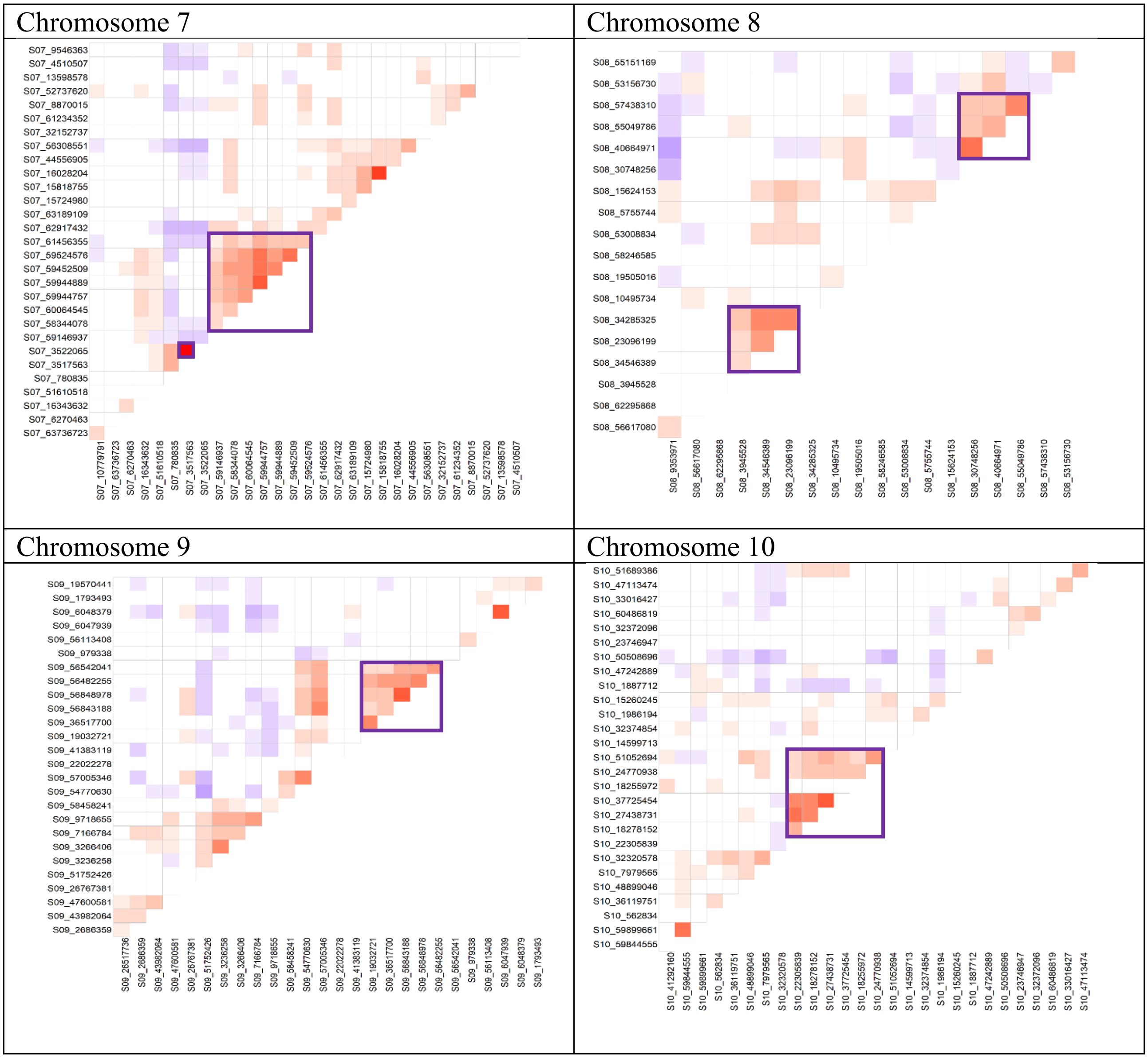

Fig. S13. Chromosome-wise correlation analysis among SNPs. Positive correlations are indicated in red, while negative correlations are shown in blue. All correlations, whether positive or negative, are considered significant at $p < 0.05$, with non-significant correlations denoted in white. Purple boxes highlight SNP blocks with positive associations.

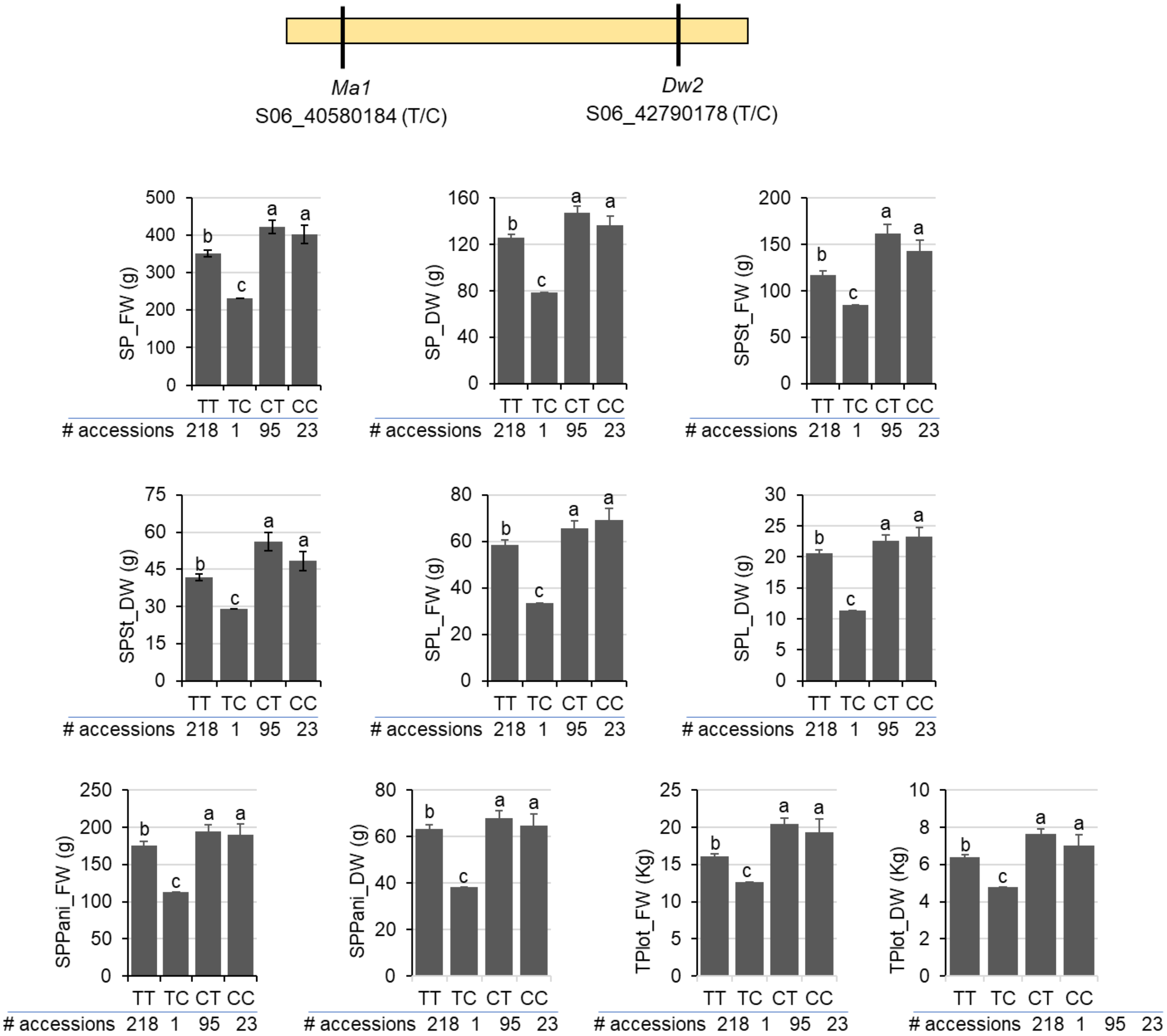

Fig. S14. Allelic variation of SNPs related to *Ma1* and *Dw2* loci and their impact on multiple biomass yield traits. Phenotypic data were obtained from the 2020 growing season. Data represents the mean and standard error of the mean for accessions carrying different allele combinations. Different letters above the bars indicate significant differences among the accessions, as determined by one-way ANOVA with Tukey's HSD post-hoc test at $p \leq 0.05$.

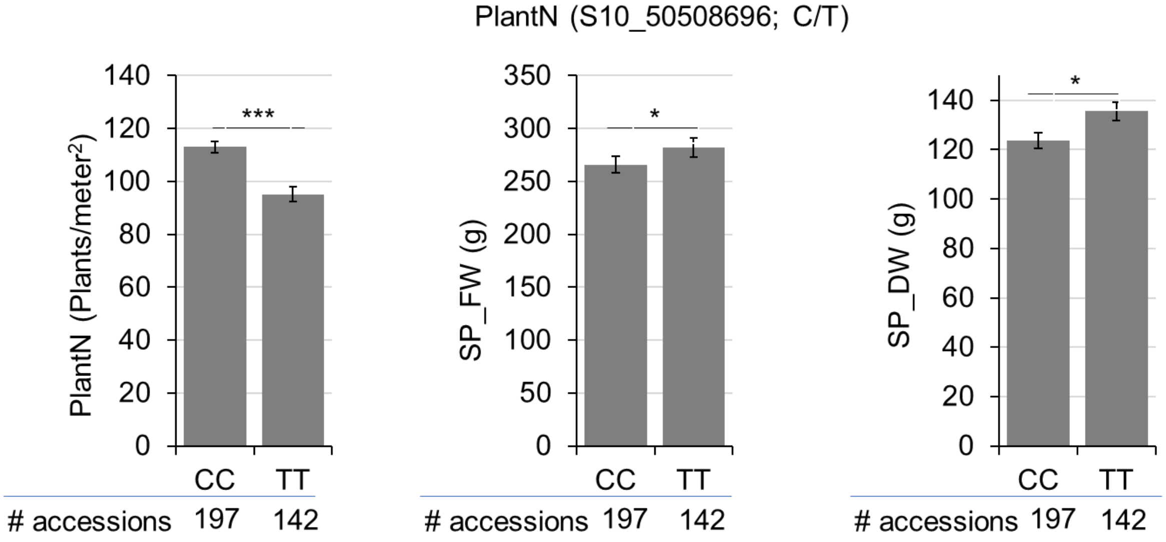

Fig. S15. Allelic variation of the locus associated with PlantN and its impact on plant biomass (SP_FW and SP_DW). Phenotypic data were obtained from the 2021 growing season. Data represents the mean and standard error of the mean for accessions carrying different allele combinations. A Student's t-test was performed to assess significant differences between groups ($p < 0.05$*, $p < 0.01$**, $p < 0.001$***, *ns*: 'not significant').

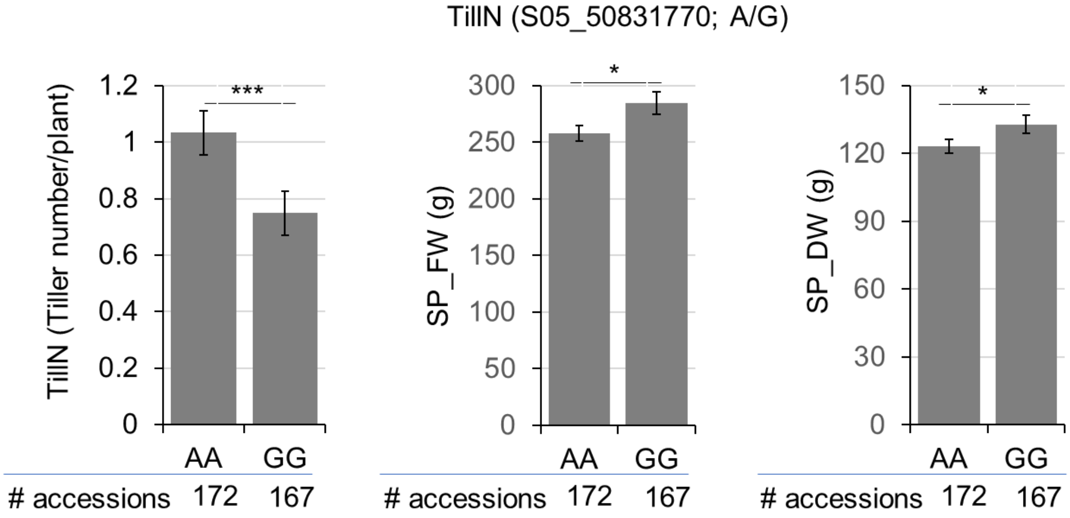

Fig. S16. Allelic variation of the locus associated with TillN and its impact on plant biomass (SP_FW and SP_DW). Phenotypic data were obtained from the 2021 growing season. Data represents the mean and standard error of the mean for accessions carrying different allele combinations. A Student's t-test was performed to assess significant differences between groups ($p < 0.05$*, $p < 0.01$**, $p < 0.001$***, *ns*: 'not significant').
